# Mylabris: a review of its biological characteristics, chemical composition, pharmacological, toxicology, pharmacokinetics, and marketed drugs

**DOI:** 10.3389/fphar.2025.1652857

**Published:** 2025-11-05

**Authors:** Qingqing Cai, Jing Yan, Xinghong Li, Lihua He, Shan Xie, Yaxin Yang, Hongwei Wu, Fangbo Zhang

**Affiliations:** ^1^ Institute of Chinese Materia Medica China Academy of Chinese Medical Sciences, Beijing, China; ^2^ Zunyi Medical University, Zunyi, Guizhou, China; ^3^ Guizhou Shenqi Pharmaceutical Co., Ltd, Guiyang, Guizhou, China

**Keywords:** mylabris, cantharidin, pharmacological, toxicity, pharmacokinetics

## Abstract

**Ethnopharmacological relevance:**

*Mylabris* (“斑蝥’’), derived from the dried bodies of the Chinese blister beetles *Mylabris phalerata* Pallas and *Mylabris cichorii* Linnaeus, which has the effect of breaking blood and chasing blood stasis (“破血逐瘀”), dispersing knots and eliminating symptoms (“散结消癥”), and attacking poison and eroding sores (“攻毒蚀疮”).

**Aim:**

This review provides the firstly comprehensive summary of mylabris, covering its biological characteristics, chemical composition, pharmacological, toxicology, pharmacokinetics, and clinical use.

**Materials and methods:**

A systematic literature search was conducted in databases (“Web of Science”, “PubMed”, “Google Scholar”, “CNKI”, and “WanFang”) using the following query (“*Mylabris phalerata* Pallas” OR “*Mylabris cichorii* Linnaeus” OR “Mylabris” OR “Banmao” OR “Cantharidin”) AND (“Pharmacology” OR “Toxicity” OR “Pharmacokinetics” OR “Marketed drugs”), to identify literature published between 2000 and 2025, focus on referring to 2015–2025. Articles with methodological defects (e.g., sample size less than 5 per group, no standardized purity detection method used), incomplete data (e.g., no access to the original literature, lack of key data values), and ethical problems (no declaration of ethical approval) were excluded. Online websites were also used, including https://ydz.chp.org.cn/#/main (Chinese Pharmacopoeia), https://www.nmpa.gov.cn/datasearch/home-index.html#category=yp (National Medical Products Administration), to obtain information on mylabris- or cantharidin-marketed drugs. Chemical structures in SMILES format were retrieved from the PubChem, and two-dimensional chemical structures were generated using ChemDraw 22.0.0.

**Results:**

The major components of *mylabris* include terpenoids, metallic elements, fatty acids, and peptides. Pharmacological research have demonstrated its anticancer, antithrombotic, and antiviral effects in preclinical study, as well as insecticidal and antifungal in agriculture. Cantharidin is considered to be the main active and toxic component, which can cause gastrointestinal, cardiovascular and respiratory toxicity if used improperly. Pharmacokinetic studies reveal that orally cantharidin predominantly accumulates in the liver and kidneys, exhibiting strong irritancy and low bioavailability. Given its therapeutic efficacy, researchers have also developed various mylabris and cantharidin-based drugs in clinical setting.

**Conclusion:**

Mylabris has been used in traditional Chinese medicine for millennia. Now, it treats various diseases and shows development potential. Future studies should focus on four key aspects: comprehensive characterization of active components, elucidation of pharmacological mechanisms, supplementation of pharmacokinetic data, and clarification of toxicological mechanisms. This paper reviews the research progress of mylabris, bridging traditional applications and modern investigations to advance contemporary research and evaluate its therapeutic potential for human diseases.

## Graphical Abstract

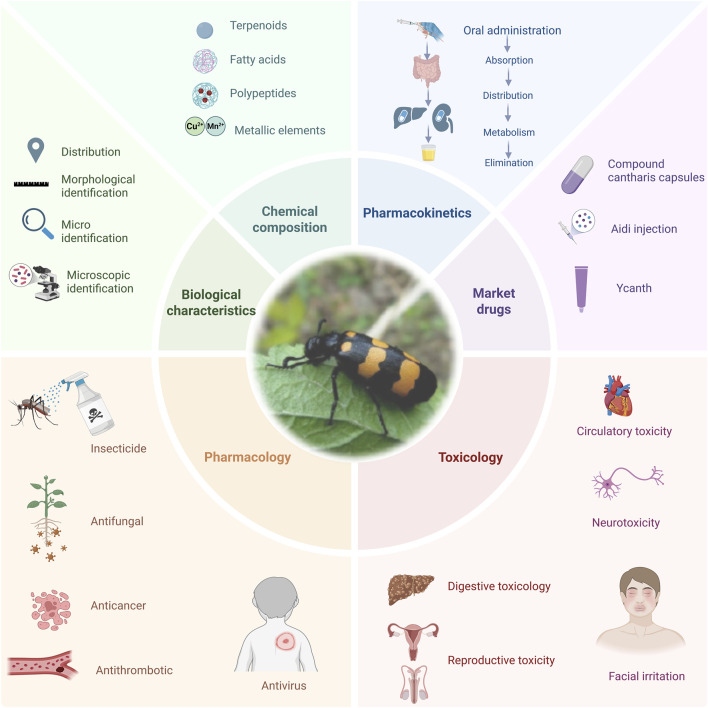

## 1 Introduction

Mylabris, a traditional animal-derived medicine, derived from the blister beetles *Mylabris phalerata* Pallas and *Mylabris cichorii* Linnaeus of the family *Meloidae*. It has been utilized in Chinese medicine since the Han Dynasty. Its therapeutic applications are documented in ancient pharmacopoeias such as *Shennong Bencao jing* and traditional medical texts of ethnic minorities, including Tibetan and Mongolian medicine (Li et al., 2024). As recorded in the *Chinese pharmacopoeia* (2020 edition), mylabris is characterized by pungent flavor, hot nature, extremely toxic and is attributed to the liver, stomach, and kidney meridians. It possesses potent effects in breaking blood stasis, dispersing hard accumulations, and counteracting toxins, making it clinically valuable for treating amenorrhea, abdominal masses, and stubborn dermatoses (Efferth et al., 2007). Modern research has revealed that mylabris contains terpenoids, fatty acids, amino acids and various metal elements. Cantharidin (CTD), the core active ingredient, is regarded as a key quality marker and exhibits antitumor (Li S. et al., 2023), antithrombotic, and antiviral properties, along with agricultural uses as a bioinsecticide and antifungal agent (Ratcliffe et al., 2011). Capitalizing on its pharmacological potential, mylabris have been formulated into various preparations, including the antitumor Aidi injection, and the topical cream Ycanth for treating molluscum contagiosum, highlighting its broad clinical prospects (Zhu et al., 2020).

However, the significant toxicity of mylabris severely limits its pharmaceutical development. Improper use may lead to severe damage to the digestive, circulatory, and reproductive systems (Fang and Du, 2018). The paucity of data regarding its absorption, distribution, metabolism, and excretion (ADME) properties presents a fundamental challenge to developing effective detoxification strategies and next-generation derivatives with improved safety profiles (Duan et al., 2021). Moreover, with the surge in market demand, wild mylabris populations are on the edge of exhaustion, while artificial breeding technology is not mature, resulting in supply-demand imbalance, the soaring price, and the proliferation of counterfeit products (Mo et al., 2014).

To address these challenges, this review provides, for the first time, a comprehensive synthesis of mylabris, encompassing its biological characteristics, chemical composition, pharmacological, toxicological, pharmacokinetics, and marketed drugs. We hope that this in-depth review could serve as a foundation for the rational development, safe clinical application, and future research on mylabris.

## 2 Biological characteristics

Mylabris belongs to the *Meloidae* family. There are more than 3,000 species in the 120 genera of *Meloidae* in the world wide, with more than 202 species in 26 genera reported in China alone (Wang, 2014). These species are distributed across most provinces and regions. Their distribution varies slightly. The vertical distribution ranges from low-altitude plains or hilly areas to high-altitude plateaus, for example, *M. phalerata* Pallas is mainly distributed in Jiangsu, Zhejiang, Hubei, Jiangxi, Fujian, Taiwan, Guangdong (Deng et al., 2017), and *M. przewalskyi* can be found at altitudes of up to 4,900 m. Additionally, the distribution of mylabris in a given area is positively correlated with the density of locusts and the types and quantities of legume crops (Mo et al., 2014). The medicinal species include *M. phalerata* Pallas and *M. cichorii* Linnaeus. Due to the high cost and limited availability, adulterated or counterfeit products are frequently encountered in the market.

Given the clinical relevance and toxicological concerns of mylabris, its accurate identification is a prerequisite for safe and effective application. The key identification methods, including morphological and microscopic, are summarized in Table 1.

**TABLE 1:** Diagnostic characteristics of *M. phalerata* Pallas and *M. cichorii* Linnaeus.

Diagnostic feature	*M. phalerata* pallas	*M. cichorii* linnaeus
Body length	1.5–3.0 cm long, 0.5–1 cm wide	1.0–1.5 cm long, 0.5–0.7 cm wide
Elytra	Dorsal with leathery elytra pair, with 3 wavy yellow-brown to red-brown stripes	Dorsal with leathery elytra, with 3 pale yellow to brown yellow transverse stripes
Inner wing	More folds, veins brown red to brown black	Few folds, veins pale yellow to yellowish brown
Tentacle	Antennal terminal segment base markedly narrower than anterior segment	Antennal terminal segment base nearly as wide as anterior segment
Setae	Setae are very much, fine spiny, more straight	Setae are less, shorter, and more twisted
Body wall fragments	Yellowish-white to tan	Pale yellow and translucent
Elytra fragments	Round, button-shaped with prominent concentric rings, the hair follicle pits left after bristle shedding are inconspicuous	The circular button-shaped concentric ring pattern is relatively small, and the concave left after the bristles fall off is obvious
Inner wing fragment	The threaded catheter-like wing veins are thick	The threaded catheter-like wing veins are relatively slender

This table is summarized from website “http://www.zhongyoo.com/jianding/3004.html” (Zhongyoo, 2012).

## 3 Chemical composition

The chemical composition of mylabris is complex and includes terpenoids, metallic elements, fatty acids, polypeptides and amino acids.

### 3.1 Terpenoids

**FIGURE 1:**
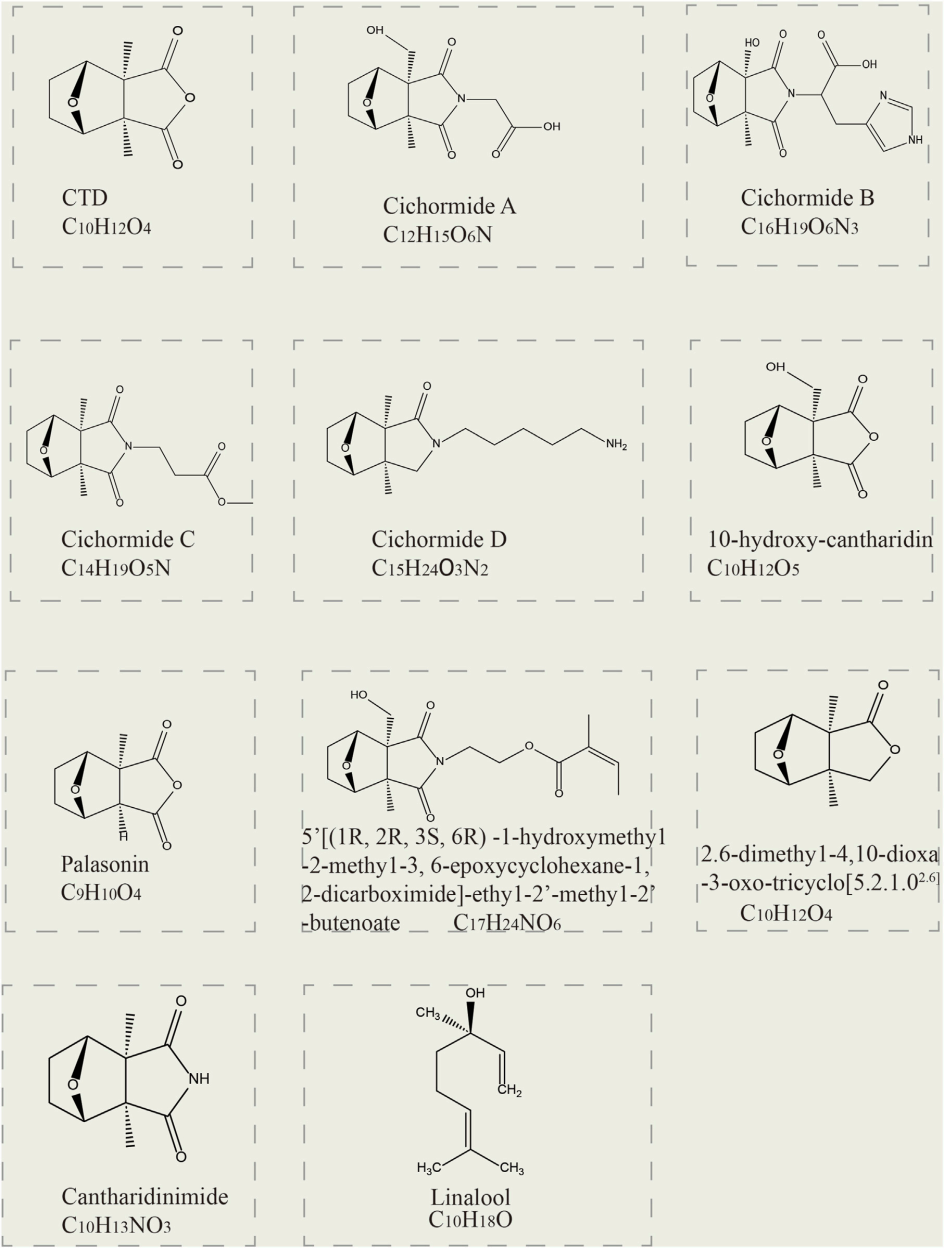
Two-dimensional structural formulas of 11 terpenoid constituents derived from mylabris.

Currently, 11 terpenoids have been found in mylabris (Figure 1). CTD, the principal bioactive terpenoid in mylabris, serves as both a quality control marker and the most extensively researched component of this medicinal insect. CTD contents in the head, foot, outer wing, and inner wing are 0.050%, 0.042%, 0.044%, and 0.024%, respectively, amounting to a total of 0.160% (Li et al., 2007; Qi et al., 2010). Conjugated CTD is the major storage form and also an important water-soluble component in mylabris. It can cause adverse clinical reactions due to its low bioavailability, high toxicity, and strong irritant. Based on the structure-activity relationship of its parent nucleus, researchers have derived a series of compounds, including sodium cantharidinate (C_10_H_12_O_4_N_a2_), demethylcantharidin (C_8_H_8_O_4_), and sodium demethylcantharidinate (C_8_H_8_O_5_Na_2_), which exhibit enhanced efficacy and less toxicity (Wang G. et al., 2018).

### 3.2 Metallic elements

The synthesis of conjugated CTD in mylabris requires specific metal elements as raw materials. The content of the same metal elements varies across different breeds and regions. This variation can serve as an important indicator to identify regions and breeds (Lou et al., 2018). The metal elements in mylabris exhibit diverse functions. For example, magnesium (Mg), calcium (Ca), potassium (K), and natrium (Na) are the raw materials to synthesize conjugated CTD. Manganese (Mn), zinc (Zn), copper (Cu), and Mg are anticancer elements. However, mylabris also contains elements, such as chromium (Cr), hydrargyrum (Hg), lead (Pb), cadmium (Cd), beryllium (Be), tin (Sn), and nickel (Ni), which could be detrimental (Yin and Jin, 2010).

### 3.3 Fatty acids, polypeptides, and other compounds

Fatty acids are significant components in mylabris. Oleic acid, linoleic acid, and octadecenoic acid exhibit potential as therapeutic agents against hepatocellular carcinoma (HCC). Palmitic acid inhibits the proliferation and migration of HCC cells by modulating glucose metabolism and reducing the membrane fluidity of HCC cells (Chai, 2023). The other fatty acids identified in mylabris include stearic acid, palmitoleic acid, linolenic acid, arachidic acid, 6-octadecenoic acid, myristic acid, and palmitic acid (Díaz-Navarro et al., 2021), there are no reports regarding the pharmacological effects of the aforementioned components (Li K. M. et al., 2023).

Mylabris also contains various peptides, including ring-(L-proline-L-alanine), ring-(R-proline-R-leucine), ring-(S-proline-R-leucine), and ring-(D-proline-L-tyrosine); nucleosides such as uracil, uridine, and hypoxanthine; aromatic compounds such as indole-3-aldehyde, indole-acetic acid, 2-piperidone, 4-hydroxyphthalid (Zeng et al., 2016), 3-phenyl-4-aza-fluorene, phenylacetaldehyde (Liu et al., 2013), and p-hydroxybenzoic acid. With the advances in modern science and technology, more components of mylabris could be discovered and serve as valuable references for its quality control.

## 4 Traditional use and modern research

### 4.1 Traditional use

**FIGURE 2:**
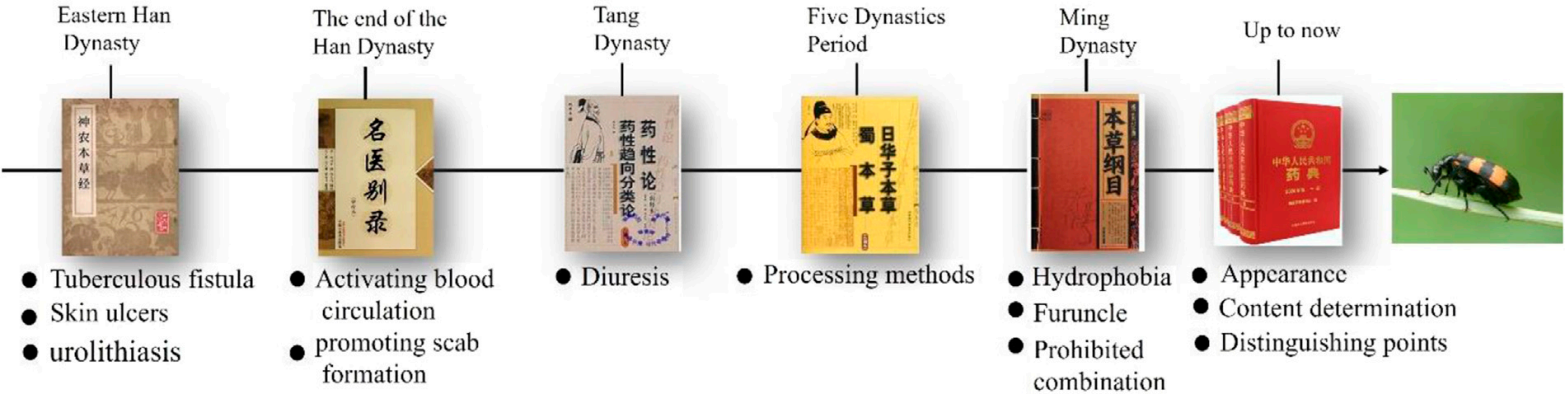
Ancient books pertaining the utilization of mylabris in China.

Mylabris has been documented in the material medica of different dynasties in China (Figure 2). Mylabris was originally recorded in the *Shennong Materia Medica* as an inferior-grade drug to treat tuberculous fistula, skin ulcers, and urolithiasis. It is also noted that mylabris should not be used in combination with *Croton tiglium* L. or *Salvia miltiorrhiza* Bunge. During the Wei and Jin dynasties, its use for activating blood circulation, promoting scab formation, and inducing abortion was documented in the *Ming Yi Bie Lu*. In the Tang dynasty, it was used for diuresis, as recorded in the *Yao Xing Lun*. In the Five dynasties period, processing methods such as the removal of wings, feet, and frying were documented in the *Ri Hua Zi Ben Cao* to prevent vomiting and diarrhea. In the Ming dynasty, mylabris was used to treat hydrophobia, furuncle, and neuralgia, and it was noted that this drug should not be used with *Glycyrrhiza uralensis* Fisch. To date, the efficacy and applications of mylabris have been detailed and revised in the *China Pharmacopoeia*, which details its appearance and shape, physicochemical characteristics, dosage and administration (Li and Chen, 2025).

Ethnopharmacological applications of mylabris vary across different ethnic groups in China. Specifically, Tibetan practitioners use it to treat food stagnation, while the Li ethnic group employs it for facial hemiplegia and tonsillitis. In Uygur traditional medicine, it is prescribed for vitiligo, eczema, pruritus, rheumatism, and erectile dysfunction. Additionally, the Hmong people utilize it to manage lymphatic tuberculosis, scrofula, and rabies. The Manchu traditionally apply mylabris against malaria. Meanwhile, the Achang, Jingpo, and De’ang ethnic groups employ it therapeutically for managing bone fractures and hemorrhagic wouns (Liu et al., 2019).

Mylabris possess notable medicinal value and is widely used in traditional Chinese medicine (TCM) formulations for treating diverse diseases (Table 2). Due to its toxicity, it is often used topically in the form of a powder (mixed with honey or vinegar) to treat carbuncles, boils, rhinitis, cough, and asthma. For oral administration, it is commonly roasted (with or without the removal of the head, feet, and wings) by cleaning or stir-frying with rice to reduce its toxicity and irritation, when treating scrofula, amenorrhea, and rabies (Ren et al., 2020). Nowadays, several prescriptions containing mylabris have been used in the form of tablet, injection, and capsules. Some prescriptions, such as Kangsaidi capsules, Aidi injection, and Ycanth, have been widely used.

**TABLE 2:** Traditional use of mylabris to treat diseases recorded in ancient books.

Preparation name	Type	Composition	Indications	References
Awei leiwan San	Powder	1. *Ferula sinkiangensis* K.M.Shen., 2. Realgar, 3. Fluorite, 4. Cinnabar, 5. Talc, 6. Chalcanthite, 7. Cinnabar, 8. *Arisaema heterophyllum* Blume*,* 9. P*aeonia lactiflora* Pall, 10. Rhinoceros Horn, 11. Mylabris*,*12. Calculus bovis	Hansen’s disease	Qian jin yi
Bading Dan	Pills	1. Olibanum, 2. Bufonis venenum, 3. Arsenic trioxide, 4. *Caryophylli* flo*s*, 5. Draconis sanguis, 6. Moschus, 7. Mylabris, 8. Chalcanthite, 9. Realgar, 10. Magnetite, 11. *Ricini* semen	Boils, carbuncles, swelling, and pain	Jing yan fang
Badou Wan	Pills	1. *Crotonis* fructus., 2. Mylabris	Deafness	Zhou hou fang
Badu San	Powder	1. Mylabris, 2. *Crotonis Fructus,* 3. Olibanum, 4. Myrrha, 5. Peucedani radix, 6. Scrophularia ningpoensis Hemsl., 7. Bovis cornu, 8. Moschus, 9. Borneol	Localized swelling with hardness	Gu jin wai fang
Bafu Wan	Pills	1. Aconiti Lateralis Radix Praeparata, 2. mylabris, 3. *Crotonis* fructus	Infantile Intestinal gas distension	Pu ji fang
Banji Wan	Pills	1.Menthae haplocalycis herba, 2. Mylabris	Scrofula	Yi xue ru men
Banmao Ding	Water	Mylabris	Neurodermatitis	Zhong yi pi fu bing xue jian bian
Banmao Fen	Powder	1. Mylabris, 2. Alumen, 3. Sulfur, 4. Plumbum 5. Oxidatum, 6. Trisulphur, 7.Synthetic borneol, 8. Arsenic trioxide, 9. Borax, 10. Moschus, 11. Glycerin	Tinea corporis	Zhong yi pi fu bing xue jian bian
Banmao Gao	Paste	1. Mylabris, 2. Colophonium, 3. Crotonis Fructus	Scrofula	Sheng hui
Banmao San	Powder	1. Mylabris, 2. M*argarita*, 3. Hydrargyrum	Multiple fistulas	Sheng ji zong lu
Banmao Wan	Pills	1. Mylabris, 2. Moschus, 3. Cinnabaris, 4. Zingiberis rhizoma, 5. *Glycyrrhiza uralensis* Fisch. ex DC., 6. Rhinoceros horn, 7. Oryzae semen	Scrofula	Sheng hui
Baoming Wan	Pills	1. *Angelica sinensis* (Oliv.) Diels., 2. Aconiti lateralis radix praeparata, 3. *Paeonia lactiflora* Pall., 4. Cinnamomi cortex, 5. Zingiberis rhizoma, 6. *Rheum officinale* Baill., 7. mylabris	Blood stasis, dyspepsia, gastrointestinal disturbance	Sheng ji zong lu
Baozhu Dan	Powder	1. Moschus*,* 2. Camphora, 3. Caryophylli flos, 4. *Crotonis* fructus, 5. Mylabris, 6. *Lycium barbarum* L	Pausimenia	Chi shui xuan zhu
Bima San	Powder	1. *Abutilon theophrasti*, 2. *Agapanthus africanus*, 3. Hippocampus, 4. *Meloe coarctatus*, 5. *Viola yedoensis* Makino*,* 6. Cuprum, 7. mylabris, 8. Realgar, 9. Arsenic trioxide	Furuncle	Yi fang lei ju
Binlang San	Powder	1. Scorpio, 2. mylabris, 3. Crotonis fructus, 4. Arecae semen, 5. Oleum sesami, 6. Phellodendri cortex, 7. Sulfur, 8. Cnidii fructus, 9. Realgar, 10. Sepiae endoconcha, 11. Amyris, 12. *Coptis chinensis* Franch, 13. Armeniacae semen amarum, 14. Sublimed mercury	Chronic shank ulcer	Zhu shi ji yan fang
Bixiao San	Powder	1. *Hibiscus syriacus* cortex, 2. mylabris, 3. *Pinellia ternata* (Thunb.) Makino, 4. Momordicae semen, 5. Arecae semen, 6. Realgar 7. Arsenic trioxide	Rheumatism, scabies, and long-standing stubborn tinea	Gu jin yi jian
Ezhang fengjinji	Powder	1. Mylabris, 2. Scolopendra, 3. Arsenic trioxide, 4. Strychni semen, 5. *Bletilla striata* (Thunb.) Reichb. f. , 6. *Rheum officinale* Baill, 7. *Strychnos nux-vomica semen*	Tinea manuum	Zhong yi pi fu bing xue jian bian
Ezhang fengyaoshui	Water	1. Pseudolaricis cortex, 2. Cnidii fructus, 3. *Hydnocarpus anthelminticus*, 4. Stemona sessilifolia (Miq.)Miq, 5. *Saposhnikovia divaricata* (Turcz.) Schischk. , 6. *Angelica sinensis* (Oliv.) Diels, 7. *Impatiens balsamina,* 8. Platycladi semen, 9. *Evodia rutaecarpa* (Juss.) Benth, 10. Zanthoxyli pericarpium, 11. Cicadae periostracum, 12. mylabris	Tinea manuum, onychomycosis, eczema, athlete’s foot	Pharmacopoeia of the People’s Republic of China

### 4.2 Modern research

**FIGURE 3:**
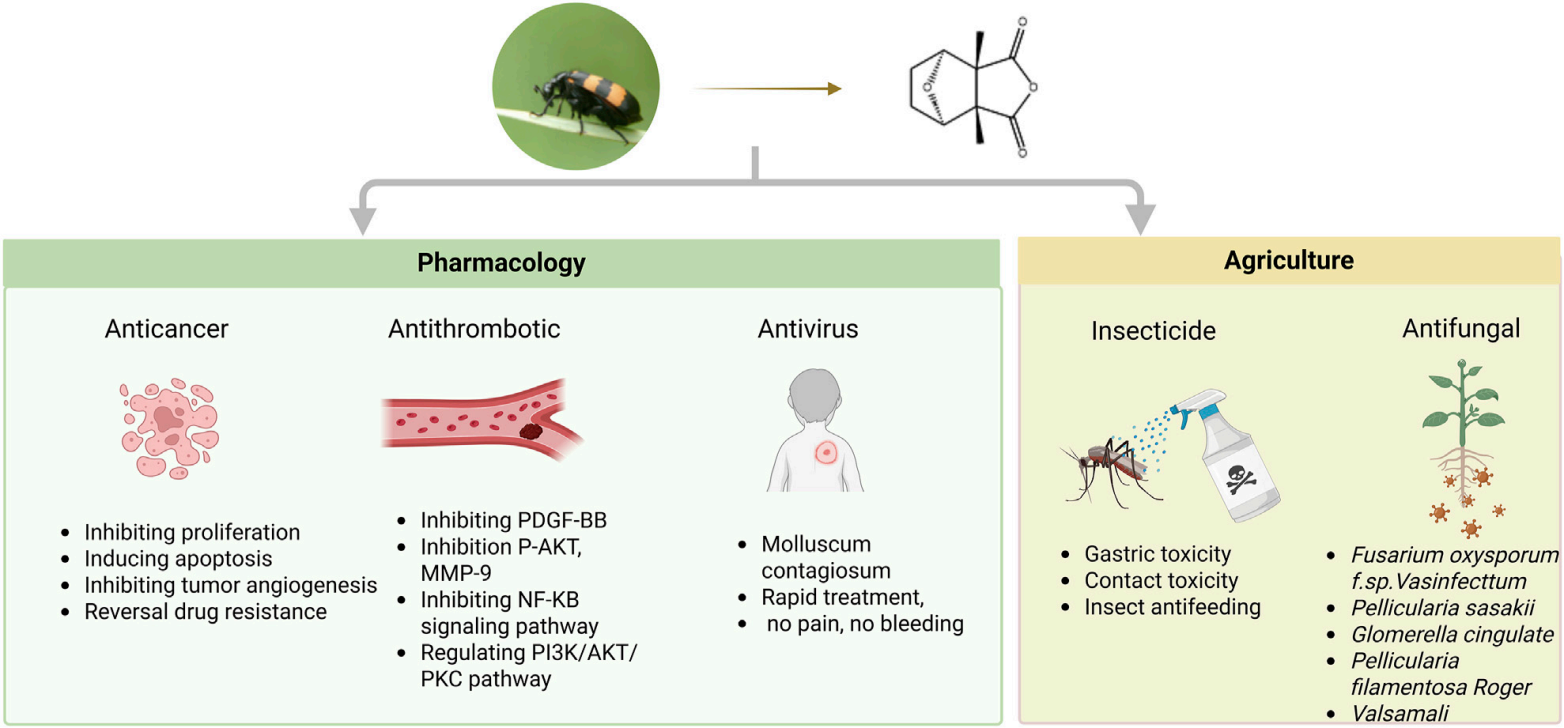
Multifunctional pharmacological properties of CTD.

Extensive studies have been conducted to determine the biological activities and pharmacological effects of mylabris extracts, particularly CTD. The currently reported activities of CTD include anticancer (Deng et al., 2013), antithrombotic (Viallard and Larrivée, 2017), antiviral (Braue et al., 2005), insecticidal, and antifungal effects (Eisner et al., 1974). Next, the pharmacological effects of CTD are summarized in Figure 3.

#### 4.2.1 Anticancer effects

The medicinal use of mylabris for cancer was first documented by Yang Shiying, a renowned physician of the Southern Song Dynasty (Safenraiter et al., 2024). In 1980, CTD was identified as an active compound against liver cancer, and subsequent preclinical studies have since demonstrated its efficacy against multiple tumor types. Research on CTD’s antitumor properties remains more extensive than its other pharmacological activities (Li et al., 2018). Table 3 summarizes *in vivo* and *in vitro* models, dosages, and mechanistic insights underlying CTD’s anticancer effects.

**TABLE 3:** Anticancer properties and mechanisms of CTD.

Cancer type	Cell lines/model	Dosage/administration/IC_50_	Mechanism of action	References
Liver cancer	HepG2, MHCC-97H, Hep3B, MHCC-97L, SMMC-7721, Huh-7 cells	0.5, 1, 1.5, 2 µM for 48 h	Downregulation of EphB4, blocking of the EphB4/PI3K/Akt signaling pathway, blocking of the EphB4/JAK2/STAT3 signaling pathway	Zhu et al. (2020)
	HepG2 xenograft models	0.1, 0.2, or 0.4 mg/kg, oral administration		
	HepG2	0, 2, 4, 5, 10, 15, 20 µM for 48 h	G2/M phase arrest, inducted of apoptosis	Le et al. (2016)
	HepG2 xenograft male BALB/c mice	0.25, 0.5, 1 mg/kg, oral administration	Cell apoptosis, immune response	Yan et al. (2023)
	LO2 cells	0, 6.25, 12.5, 24, 50, and 100 µM for 24 h	Inhibition of ERS (GRP78, ATF4, PERK, p-PERK, XBP1–1, and CHOP), induction autophagy (LC3, Beclin-1, Atg3, Atg4A, Atg4B, and Atg7), induction of apoptosis (Bax/Bcl-2 and caspase-3)	Liu et al. (2020)
	HEK293T cells, L02 cells, HepG2 cells	0.5, 1, 2 ug/mL	Downregulation of MDR1 gene expression	Zheng et al. (2008)
	SMMC-7721, HepG2 cells	0, 5, 10, 15 µM for 24 h	Induction of DNA damage, enhancement of chemotherapy sensitivity via KDM4A-dependent demethylation of histone H3K36	Wei et al. (2022)
	HepG2 xenograft models	1.34, 2.67 mg/kg, oral administration	ERS, autophagy, and apoptosis	Wu et al. (2015)
Gastric cancer	MGC803, BGC823	0, 5, 10 µM for 0, 12, and 24 h	Downregulation of CCAT1, Akt, and MDM2, upregulation of the PI3K/Akt signaling pathway	Song et al. (2020)
	SGC-7901, BGC-823 cells	2.5, 5, 10, 20, 40, 80 µM for 24, 48, and 72 h	G2/M phase arrest (downregulation of cyclin A and B and CDK1, upregulation of p21); induction of apoptosis (upregulation of caspases-7, -8 and -9; activated caspase-3, PARP, and Bad, downregulation of Bcl-2 and Bid)	Zhang et al. (2014)
Colorectal cancer	HCT116, SW620	HCT116 24 h: IC_50_ = 12.4 ± 0.27 µM; HCT116 48 h: IC_50_ = 6.32 ± 0.2 µM; SW620 24 h: IC_50_ = 27.43 ± 1.6 µM; SW620 48 h: IC_50_ = 14.30 ± 0.44 µM	Inhibition of S100A4 and MACC1	Schöpe et al. (2023)
	Colo 205 cells	IC_50_ = 20.53 µM	G2/M phase arrest, downregulation of CDK1 activity, decrease Cyclin A, Cyclin B, CDK1, pro-caspase-8, pro-caspase-9, and Bcl-2, increase CHK1 and p21 protein levels, ROS production	Huang et al. (2011)
	HCT116 cells	1, 5, 10, 30, and 50 µM for 24 h	Inhibition of HSP70 and BAG3, downregulation of BCL-2 family proteins	Kim et al. (2013)
	HCT116 cells	0, 10, 20, and 30 µM or 48 h	Inhibition of proliferation and migration, promotion of apoptosis	Hou et al. (2024)
Pancreatic cancer	PANC-1, CFPAC-1	0.1, 0.3, 1, 3, and 10 µM for 24 h	G2/M phase arrest, DNA damage, repression of JNK, ERK, PKC, P38, and NF-κB	Xu et al. (2018)
Lung cancer	A549 cells	0, 1, 3, 10, 30, and 100 µM for 24 h	Downregulation of MMP-9 and MMP-2, inhibition of the PI3K/Akt signaling pathway	Kim et al. (2013)
	H460 cells	0, 5, 7.5, 10, 15, and 30 µM for 24 h and 48 h	Increased caspase-3 and -8, cytochrome c, Bax, AIF, calpain 2, and XBP-1 levels, inhibition of Bcl-xL and calpain 1	Hsia et al. (2014)
	NCI-H460 cells	0, 5, 10, 15, and 20 µM CTD for 24 h and 48 h	Reduction of BRCA-1, 14-3-3σ DNA-PK and MGMT	Hsia et al. (2015)
	H460 cells	10 µM for 24 h	DNA damage, cell cycle progression, and apoptotic cell death	Hsia et al. (2015)
	A549 cells	1 µM	Apoptosis (downregulation of Bcl-2, upregulation caspase-3 and Bax levels), autophagy (downregulation of p62, upregulation 1A/1B light chain 3B and Beclin-1)	Liu et al. (2018)
	NCI-H460 cells	0, 1, 2.5, 5, 7.5, and 10 µM for 24 h and 48 h	Reduction in FAK, GRB2, Ras, TIMP2, TIMP1, ROCK1, PI3K, IRE1α, MKK7, p-AKT, p-JNK1/2, p-p38, p-ERK1/2, iNOS, COX-2, NF-κB p65, UPA, and MMP-1, -2, -9, 13, PI3K, AKT, UPA, p38, JNK and ERK	Hsia et al. (2016)
	A549, H460, and H358 cells	2.5 μM for 24 h	Inhibition of PP5 induces apoptosis	Hsieh et al. (2017)
	H460 xenograft	20 mg/kg, every 3 days, oral administration		
Acute myeloid leukemia	HL-60	IC_50_ = 6.21 mM (72 h)	G2/M phase arrest, downregulation of cyclin E, cyclin B1, and CDK2, upregulation of p27 and p53, induction of Nur77	Yu et al. (2020b)
	Kasumi-1	IC_50_ = 8.00 mM (72 h)		
	OCI-AML3	IC_50_ = 28.70 mM (72 h)		
	HUVECs (normal cell)	IC_50_ = 75.63 mM (72 h)	\	
	K562	IC_50_ = 28.23 µM (24 h), 27.63 µM (72 h)	Induction of mitotic arrest, DNA damage, downregulation of BCR-ABL	Sun et al. (2016)
	K562R	IC_50_ = 54.42 µM (24 h) IC_50_ = 1.34 µM (72 h)		
Prostate cancer	DU145, LNCaP	0, 0.25, 0.50, and 1 μM for 18 h	Downregulation of c-FLIP and upregulation of DR-5	Nazim et al. (2020)
	PC-3	1, 2.5, 5, 10, 15, 20 μM for 24, 48 and 72 h	\	Lou et al. (2014)
Breast cancer	MDA-MB-231	0, 0.1, 0.5, 1, and 2 µM for 24 h	Downregulation of EGFR, GLUT1, MCT4, and MCT1	Pan et al. (2019)
	MCF-7			
	Xenograft model	Injected intraperitoneally, 0.2 mg/kg/day, 0.5 mg/kg/day	\	
	MDA-MB-231	0, 5, 10, 15 μM	Inhibition of EGFR-mediated STAT3 and AKT, induction of apoptosis (caspase-3, caspase-8, and PARP1)	Chun et al. (2018)
Breast cancer	MDA-MB-231	2.5, 5, 10, and 20 mM for 48 h	G2/M phase arrest, decrease in MEK, ERK, MAPK, JNK, MMP-9, and MMP-2	Gu et al. (2017)
	MDA-MB-231 cell xenograft model	20 or 40 mg/kg daily for 3 weeks	\	
	MCF-7 cells, MDA-MB-231 and HBL-100	0.8, 1.6, 3.2, 6.4, and 12.8 μg/mL	Inhibition of MCM7, E2F1, PTEN, p21	H. Zhang and Yan (2015)

##### 4.2.1.1 Inhibition of proliferation

CTD inhibits the proliferation of tumor cells by blocking different cell cycles. CTD blocks the G2/M phase in breast cancer cells by inhibiting the activation of mesenchymal epithelial transition factor (Met)/Sarcoma (Src)/protein kinase B (Akt 2) (Du, 2019) and reducing the expression of matrix metalloproteinase (MMP)-2 and MMP-9 (Gu et al., 2017). It also inhibits the proliferation of human gastric cancer cells SGC-7901 and BGC-823, blocks the G2/M phase. The mechanism may be related to activation of the caspase cascade and/or induction of apoptosis by regulating Bcl-2 family proteins (Zhang et al., 2014). Moreover, CTD can block human malignant melanoma A375 cells in the G0/G1 phase, likely by interfering with the extracellular regulated protein kinases (ERK) and AKT signaling pathways (Liu et al., 2018). The cell cycle also affects the radiosensitivity of tumor cells, with cells being most sensitive in the G2/M phase, less sensitive in the G0/G1 phase, and least sensitive in the late S phase. A study on pancreatic cancer indicated that CTD could enhance cellular radiosensitivity by driving cancer cells out of the quiescent G0/G1 phase and arresting the cell cycle in the G2/M phase (Xu et al., 2018). Similar results were obtained for acute myeloid leukemia (AML) (Yu Z. et al., 2020) and Lung cancer (Zhang, 2021). So, CTD exhibits broad-spectrum antitumor activity by arresting the cell cycle at different phases across multiple cancer types.

##### 4.2.1.2 Induction of apoptosis

The Bcl-2 family and caspase family are key molecules in the cell apoptosis pathway (Huang et al., 2011; Le et al., 2016). The combination of CTD and pemetrexed can inhibit the growth-inhibition rate of HCT116 colon cancer cells, reduce the expression of pro-caspase-3, and increase the expression of cleaved-poly ADP-ribose polymerase (PARP) (Sui et al., 2018). CTD downregulates epidermal growth factor receptor (EGFR) through mir607, and blocks the phosphatidylinositol 3-kinase (PI3K)/AKT/mammalian target of rapamycin (mTOR) and extracellular regulated protein kinases (ERK)/mitogen-activated protein kinase (MAPK) signaling pathways to inhibit the proliferation of BT474 and MDAMB-468 breast cancer cells (Yang et al., 2023). In liver cancer, CTD upregulates the mRNA expression of TP53, inhibits the activation of the PI3K/Akt signaling pathway (Wang et al., 2022), and enhances DNA damage and inhibits the proliferation of HepG2 and SMMC-7721 cells (Wei et al., 2022). In human bladder cancer, CTD induces the apoptosis of T24 and RT4 cells by increasing the expression of caspase-9/7/3, and regulates the ER stress pathway through the calcium/protein kinase C (PKC) pathway (Su et al., 2015). In oral cancer, CTD inhibits the proliferation of SAS, CAL-27, and SCC4 cells by increasing the expression of Bax, and Bid proteins, and decreasing Bcl-2 protein (Su et al., 2016). Additionally, CTD is used to inhibit the proliferation of HL-60 leukemia cell. The mechanism may be related to blocking the G2/M phase of the cell cycle and promoting the activation of caspase-8 and PARP (Yu Z. et al., 2020). To sum up, CTD exerts potent pro-apoptotic effects across multiple cancer types by modulating key apoptotic regulators and critical signaling pathways. Its ability to synergize with chemotherapeutics (e.g., pemetrexed) and induce DNA damage highlights its potential as a combinatorial antitumor agent.

##### 4.2.1.3 Inhibition of tumor angiogenesis

In 2014, it was first confirmed that CTD could inhibit the proliferation, migration, and tube formation in human umbilical cord vein endothelial cells (HUVEC) in a dose-dependent manner (Wang et al., 2015). In breast cancer, upon tube formation and in the rat aortic ring assay, CTD could decrease the number of completely formed tubes and reduce the density and length of vascular sprouting. These findings show that CTD can inhibit breast cancer angiogenesis (Pan et al., 2019; Li et al., 2020).

**FIGURE 4:**
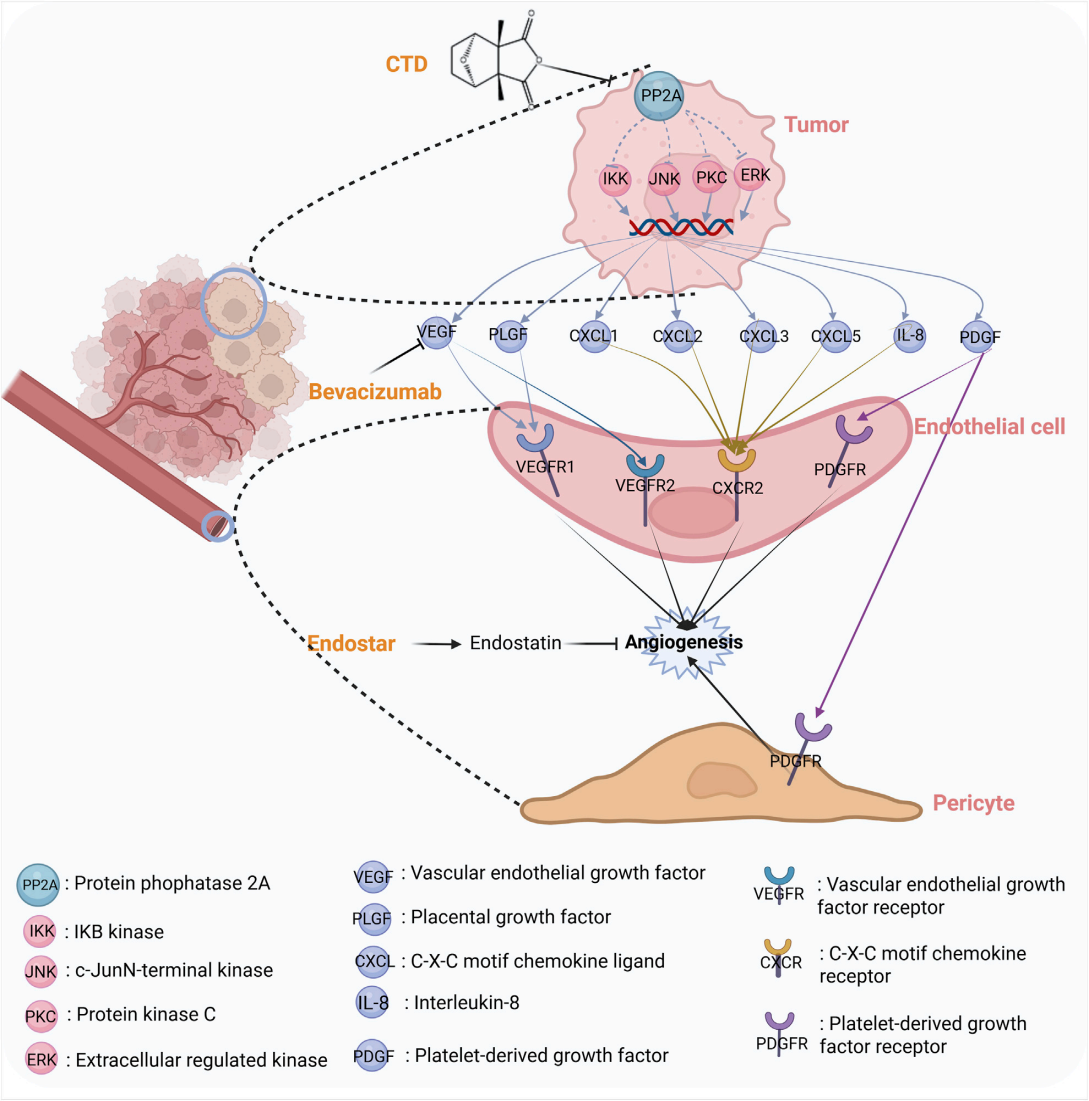
The synergistic mechanism between CTD and antiangiogenic drugs.

Several studies have also indicated that CTD may exert a pro-angiogenic effect on tumors. In pancreatic cancer, a study demonstrated that CTD significantly increased the size of transplanted tumors in nude mice. Consistent findings have also been reported in xenograft models of lung and colon cancers, indicating that CTD might promote tumor growth by enhancing angiogenesis in these malignancies. CTD can increase the levels of angiogenic factors, including vascular endothelial growth factor (VEGF), IL-6, IL-8, and TNF-α, and upregulate the expression of angiogenesis-related genes, including IL-8/C-X-C Motif Chemokine Ligand 8 (CXCL8), CXCL1/growth-related oncogene (GRO) -α, VEGF/VEGFA, granulocyte-macrophage colony-stimulating (GM-CSF)/colony stimulating factor 2 (CSF2), and plasminogen activator urokinase receptor (PLAUR)/uPAR at both the mRNA and protein levels. These findings suggest that CTD can stimulate tumor growth and potentially tumor vascular remodeling (Xu, 2019). Therefore, the antitumor effect of CTD may be masked by its angiogenic effect. However, the proangiogenic effects of CTD can be antagonized by anti-angiogenic drugs and kinase pathway inhibitors, such as, Bevacizumab, and Endostar, which exhibit significant synergistic antitumor effects. Figure 4 summarizes the synergistic mechanism of CTD and anti-angiogenic drugs. These results indicate that the clinical use of CTD to treat tumors should be conducted in conjunction with antiangiogenic therapy (Xu, 2019).

##### 4.2.1.4 Reversal of drug resistance

Imatinib is a highly specific and efficacious tyrosine kinase inhibitor that is used to treat chronic myeloid leukemia. However, imatinib resistance poses a significant challenge, limiting its clinical use. CTD can arrest cell cycle at the mitotic phase, trigger DNA damage, and downregulate B-cell receptor (BCR)-ABL protein expression, thereby overcoming imatinib resistance (Sun et al., 2016). Similarly, multidrug resistance (MDR) is a major obstacle in treating HCC. A study have demonstrated that CTD can effectively inhibit P-glycoprotein expression, mRNA transcription, and MDR1 promoter activity, suggesting the role of CTD as a novel and potent agent that can reverse MDR (Zheng et al., 2008).

Norcantharidin (NCTD), 7-oxabicyclo [2.2.1] heptane-2,3-dicarboxylic anhydride, is a demethylated analog of CTD that can reverse resistance and enhance the sensitivity to antitumor agents in various human cancers (Chen et al., 2009; Hsieh et al., 2013). NCTD exhibits enhanced anticancer potential and fewer side effects compared with CTD (Pan et al., 2020). Preclinical studies have demonstrated that NCTD can reverse chemotherapeutic resistance in cancer through multiple molecular mechanisms, including the induction of apoptosis, impairment of cancer cell stemness, and blocking of mitotic (Zeng et al., 2024).

Synthesis of the available evidence suggests that CTD and NCTD exhibit potential in reversing drug resistance. However, the scarcity of clinical trials, combined with the lack of established safe dosages, optimal timing, and well-defined combination strategies, indicates that further research is needed before this approach can be translated into clinical practice.

##### 4.2.1.5 Increasing leukocyte

CTD exerts antitumor effects without causing significant damage to the immune organs or hematopoietic factors in mice. It activates the JAK2-STAT5 signaling pathway in the bone marrow cells of mouse, thereby promoting their proliferation and differentiation while upregulating peripheral blood cell levels and decreasing myelosuppression (Li, 2023).

NCTD is the only anticancer drug that can increase white blood cell counts in a clinical setting (Mo et al., 2018). In the cyclophosphamide-induced model of leukopenia, NCTD restored hematopoietic function by promoting the G0/G1 phase of bone marrow cells to enter the S phase and G2/M phase, leading to recovery from DNA damage. At the molecular level, NCTD can downregulate the expression of BAX protein, upregulate the expression of BCL-2 protein, and decrease the ratio of BAX/BCL-2 (Zheng et al., 2015). In a clinical setting, a patient took NCTD tablets during chemotherapy, this decreased incidence of adverse effects including leukopenia, granulocytopenia, and emesis (Shen et al., 2013; Xiao et al., 2016). This study suggests that an optimal anti-cancer strategy should evolve from the conventional “fighting fire with fire” approach to a dual action paradigm that combines tumor suppression with system restoration.

#### 4.2.2 Antithrombotic effects

Mylabris contains abundant trace elements, and its fibrinolytic proteins demonstrate thrombolytic activity with dose-dependent *in vitro*. Furthermore, CTD demonstrates inhibitory effects on platelet aggregation, release, and spreading, potentially mediated through modulation of the PI3K/Akt/PKC signaling pathway (Guo et al., 2023). Further studies revealed that CTD could inhibit the platelet-derived growth factor-BB (PDGF-BB)-induced proliferation and migration of rat thoracic aortic vascular smooth cells (VSMCs), attenuating Lipopolysaccharide (LPS)-induced vascular inflammation (Qiu et al., 2019a; Qiu et al., 2019b). Given the close interplay between thrombosis and inflammation (e.g., in atherosclerosis), CTD’s dual capacity to suppress vascular inflammation and platelet activation may offer distinct therapeutic advantages against atherothrombosis.

#### 4.2.3 Antiviral effects

The antiviral effects of mylabris are attributed to its high toxicity, which aligns with the TCM theory of “fight poison with poison” (Pan et al., 2019). Molluscum contagiosum (MC) is an increasingly common skin infection caused by MC virus, a member of the poxvirus family. Its prevalence ranges from 2% to 10% in children (Braue et al., 2005). The use of CTD in the treatment of MC has been documented since the 1950s (Moed et al., 2001). The advantages of CTD over other treatments include a rapid treatment time, minimal pain at the time of application, and no bleeding. CTD is a commonly used and efficacious therapy for MC, which is generally well tolerated and associated with high rates of parental satisfaction (Moye et al., 2013). Subsequent studies have demonstrated that the combination of CTD with podophyllotoxin and salicylic acid, typically following, is highly effective removing plantar warts, with 100% success rate (Vakharia et al., 2018). This finding demonstrates the potential efficacy of the combination therapy in treating plantar warts. However, the safety and efficacy of combination therapies in younger children have not been fully described. Therefore, well-designed, randomized controlled trials with adequate blinding, control groups, and follow-up periods, as well as valid and reliable outcomes, are necessary to determine optimal protocols for CTD-based treatments (Vakharia et al., 2018).

#### 4.2.4 Agricultural application

Bioinsecticides utilize naturally derived organisms or their metabolites for pest control, exhibiting superior target specificity, low non-target toxicity, and environmental compatibility. Growing concerns over chemical pesticide safety have positioned mylabris and other bioactive natural compounds as promising candidates for next-generation bioinsecticide development.

Mylabris has insecticide effects as documented in ancient texts. However, the methodology of its application has not been documented. In 1937, *Goernitz* reported that microgram quantities of CTD could kill *Phyllopertha horticola*, *Lymantyia mornachl*, and *Pyrrhocoris apterus* among other insects. In 1974, Carrel and Eisner discovered that ants exhibited a significant antifeedant response to CTD (Eisner et al., 1974). In 1992, Frenzel reported that CTD had trapping effect on dipteran *Ceratopgonidae*, *Anthomyiidae* and other insects. In 2008, studies have demonstrated that contact-killing effect of CTD on agricultural pests, including the brown planthopper and diamondback moth. On Lepidopteran insects such as *Plutella xylostella* (Huang et al., 2015), CTD exerts strong stomach toxicity, and antifeeding effects upon contact (Zhang et al., 2000). Subsequent studies have shown the toxic effects of CTD exerts on pests, including *Agrotis ipsilon*, *Nilaparvata lugens* Stal, *Sogatella furcifera*, and *Bambusiphaga furca*. Mukaria pallipes was found to be the most sensitive and *A. grotis* to be the least sensitive to CTD (Eisner et al., 1974). Generally, CTD exhibits toxic effects on numerous pests, mainly through gastric toxicity, contact action, and antifeeding effect (Eisner et al., 1974; Pradhan et al., 2024).

CTD also exerts potent antifungal effects against several plant pathogens, including Fasarium oxysporum f. sp.vasinfecttum, Pellicularia sasakii, Sclerotinia sclertiorum, Glomerella cingulate, Pellicularia filamentosa Roger, and Valsamali (Cao et al., 2008). However, these findings have been reported based on basic experimental research, and no pesticides are being sold commercially.

The safety of biological pesticides cannot be ignored. CTD is a pesticide that has weak mobility in soil. When it reaches equilibrium in the soil-water system, it is mainly adsorbed by the soil phase, which will not pollute to the surrounding environment due to rapid degradation. Therefore, CTD is an ideal pesticide with low residue, making it environmentally friendly (Cui et al., 2009).

Fundamental studies have identified CTD as a broad-spectrum, highly efficient, and environmentally friendly bioinsecticide with significant potential for green agriculture. However, translating laboratory findings to large-scale agricultural applications requires further evaluation of its biosafety, target specificity, and environmental stability. Future research should integrate multidisciplinary approaches, including biotechnology, nanotechnology, and ecotoxicology to facilitate its industrial development.

## 5 Toxicology

**FIGURE 5:**
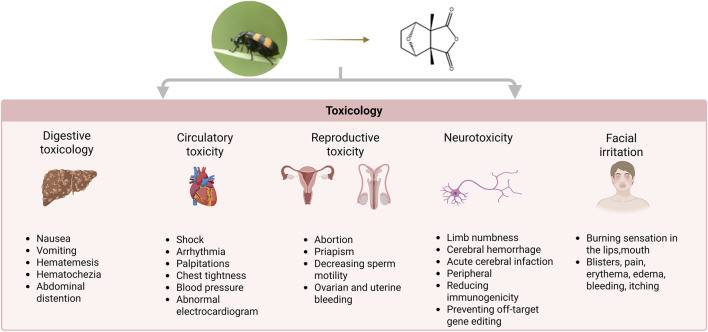
Comprehensive toxicological profile of CTD: multiorgan adverse effects.

In the Chinese Pharmacopoeia, mylabris has been recorded to be extremely poisonous. Due to the lack of knowledge about its pharmacological effects, dosage, and toxicity, and lax drug management among users, there have been cases of toxicity due to multiple doses and accidental ingestion. The different administration routes and dosage forms of mylabris will affect the occurrence of toxic reactions. Mylabris poisoning mostly results from oral administration. There are differences in toxic reactions when decoctions and powders are used. The average toxic dose is 3.91 g in decoctions, with the minimum dose being 0.1 g and the maximum dose being 60 g. Mylabris toxicity generally occurs immediately after ingestion of the oral decoction. Sometimes, it occurred at 1 h after ingestion and led to death within 6 days. However, the intensity of toxicity is higher in the powder form, and is speculated to be related to the sublimation of CTD when exposed to high temperatures during decoction. Poisoning has also occurred in individuals coming in contact with external applications and during processing. Therefore, adequate protection must be ensure during preparation and use to avoid episodes of poisoning. In-depth studies have reported CTD to be the toxic component of mylabris. The characteristics of CTD poisoning are summarized in Figure 5 (Zhang et al., 2020a; Zhang, 2022).

### 5.1 Digestive toxicity

The digestive toxicity of CTD is primarily characterized by digestive tract damage and liver failure (He et al., 2022). The principal symptoms include nausea, vomiting, hematemesis, abdominal distention, and hematochezia. Although the symptoms are commonly assumed to be linked to acute stress, the precise mechanism remains unclear.

**FIGURE 6:**
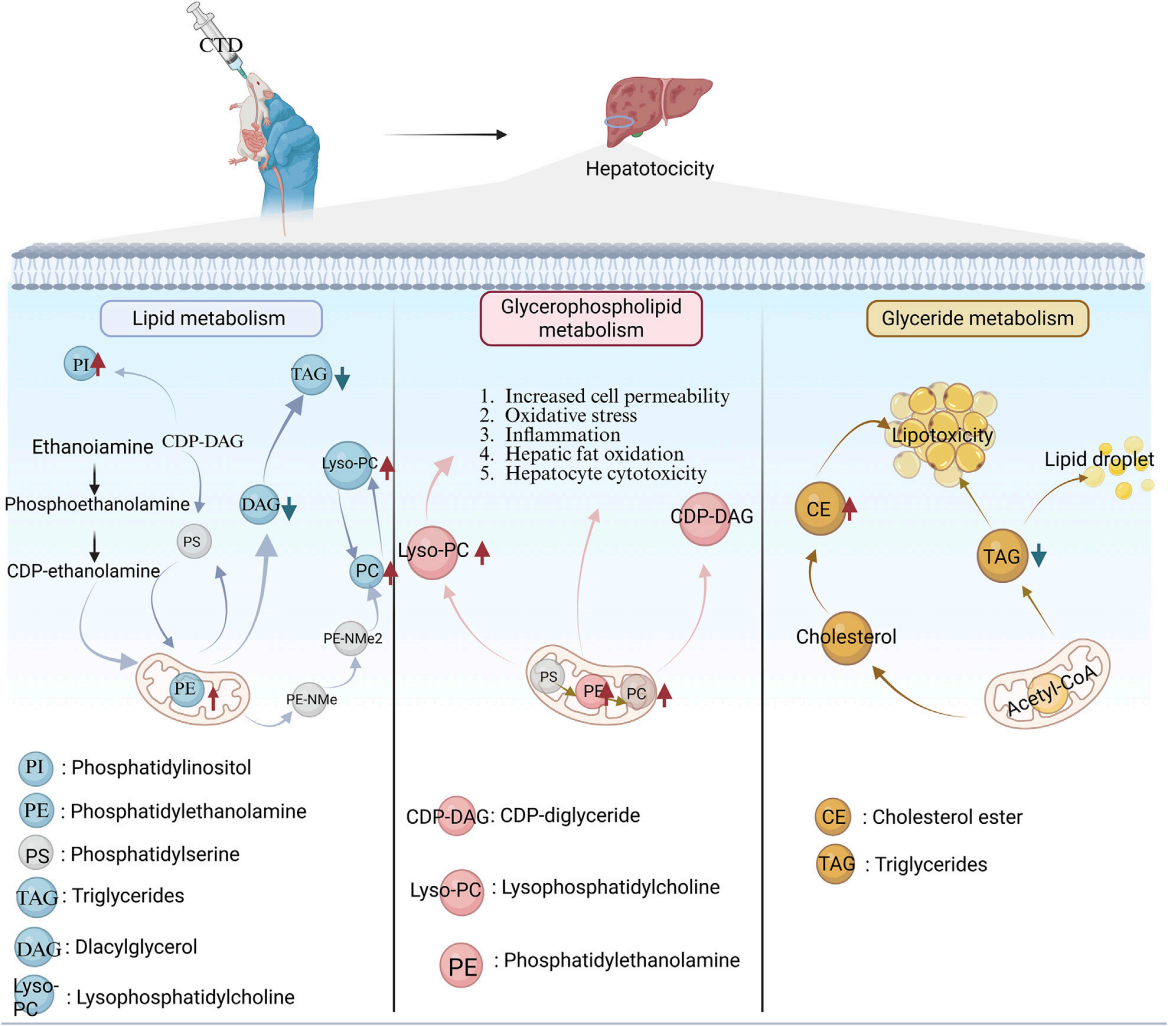
Three metabolic pathways of CTD-induced liver injury. Red upward arrows represent metabolite upregulation; Blue down arrows represent metabolite downregulation.

The liver is the primary target organ in CTD-induced digestive toxicity, with inflammatory cell infiltration and focal necrosis being the predominant pathological manifestations of CTD-induced hepatotoxicity (Yu Y. L. et al., 2020). Biochemical markers, including aspartate aminotransferase, alanine aminotransferase, and alkaline phosphatase, are significantly elevated in animal models, indicating liver injury (Cheng et al., 2022). CTD-induced hepatotoxicity is associated with following 3 metabolic pathways (Figure 6): lipid metabolism, glycerophospholipid metabolism, and glyceride metabolism (Li et al., 2024). Further studies have revealed that liver injury following CTD is associated with ER stress, autophagy, apoptosis, and metabolism (He et al., 2024).

### 5.2 Cardiotoxicity

The heart is the primary target organ in circulatory system-related toxicity caused by CTD. With sinus tachycardia being the most common symptom (Wang G. et al., 2018). Pathological changes, including myofibrillar aggregation, fibroinflammatory response, and subepicardial hemorrhage have been confirmed in the tissue samples of the heart exposed to CTD. The mechanism of myocardial injury may be related to the inhibition of VEGF by CTD, based on changes in protein expression after poisoning (Zhang et al., 2020b). Further studies have revealed that CTD may inhibit the PI3K signaling pathway, leading to a reduced neovascularization and induction of myocardial injury (Zhang et al., 2020c).

### 5.3 Reproductive toxicity

CTD, as an “aphrodisiac”, has a long history of use in Europe (Moed et al., 2001). Due to its excitatory effects on reproductive organs, it often causes ovarian and uterine bleeding or abortion in women, as well as priapism and reduced sperm motility in men during poisoning (West and Krychman, 2015). CTD-induced testicular injury involves multiple targets. CTD may inhibited expression of PI3K, AKT and BCL-2, while promoting expression of the proapoptotic proteins Bax and Caspase 3 in testicular injury (Liu R. et al., 2024). Further studies have revealed that CTD may upregulate LC3 and Beclin1 expression, while downregulating P62 and mTOR/pmTOR in the testicular tissue of mouse, leading to excessive autophagy. Furthermore, CTD can markedly diminish ZO-1, CX-43, and testosterone, indicating that it impairs the blood-testis barrier in mouse (Xiao et al., 2024).

### 5.4 Neurotoxicity

There is a paucity of reports related to poisoning of the nervous system by CTD. The primary causes of neurological impairment are acute ischemia and hypoxia. Bederson method has been used to evaluate the neurological deficit score of rats exposed to CTD, and the results demonstrate a positive correlation between the neurological deficit score and CTD dose. Previous studies have demonstrated that CTD poisoning can result in acute cerebral infarction, cerebral hemorrhage, and other focal neurological deficits, as well as peripheral neuropathy, including peripheral facial paralysis and limb numbness (Zhang et al., 2020a). Future investigations should focus on constructing complete dose-toxicity profiles to establish precise safety thresholds, thereby generating toxicology data to guide clinical practice - especially for optimizing long-term treatment safety.

### 5.5 Facial irritation

After the ingestion of CTD, a burning sensation in the lips, mouth, and pharynx occurs within minutes. Subsequently, blisters form, causing difficulty in swallowing, and abdominal cramps, hematemesis, and vomiting have also been noted. Blisters formation, pain, erythema, edema, bleeding, itching, and post-inflammatory hyperpigmentation occur in 6%–46% of patients (Zhang et al., 2018; Pradhan et al., 2024).

### 5.6 Detoxification

The management of CTD poisoning is primarily supportive, as no specific antidotes are currently available (Li et al., 2022). If accidentally exposed to CTD or ingested, the following measures may help mitigate its toxic effects: for local exposure, the affected area should be washed immediately with acetone, ether, soap, or alcohol to dissolve and dilute the toxin. The affected area should subsequently be washed thoroughly with soap and water. Topical steroids may be applied to intact skin if symptoms are present (Karras et al., 1996). Several supportive measures may be used. If CTD is taken orally, patients should drink an adequate quantity of water, while avoiding foods that high in fat (such as milk), as these can increase CTD absorption. Vomiting is not recommended as it can re-expose CTD to the mouth and trachea (Moed et al., 2001).

It is crucial to deepen toxicological and pathological studies on CTD to elucidate its mechanisms of toxicity. Such research will provide valuable insights for guiding its safe clinical use, developing antidotes for poisoning, and improving *postmortem* identification.

## 6 Pharmacokinetics

In order to improve the pharmacokinetic study of mylabris, research have used a variety of methods. The pharmacokinetic parameters of mylabris’ ethanol extract have been determined using multipoint dynamic measurement combined with assessments of animal mortality and drug accumulation. The intraperitoneal injection of mylabris ethanolic extract (75% ethanol) was a two-compartment model with first-order kinetic elimination, and the median lethal dose (LD_50_) was 344 mg/kg (Song, 2014). However, this pharmacokinetic study utilized acute mortality as an indicator in animal models. It is crucial to not only consider the differences in physiology and pathology between humans and animals, but also the significant differences between the oral and intraperitoneal routes of drug administration. Objectively, the pharmacokinetic parameters derived from the toxicodynamic method merely serve as a general estimate, providing only a reference point for drug application.

The WinNonlin 6.4 software was used to fit the concentrations and time of CTD in rat plasma following the administration of different doses of the aqueous extract of the mylabris compound (AEMC, 20 g of Mylabris powder was refluxed with 400 mL 75% ethanol for 1.5 h twice and was afterwards filtered). The time to peak concentration was determined to be 0.17 h for all tested doses of AEMC, indicating the rapid absorption of CTD into the bloodstream following its oral administration. Additionally, the volume of distribution (Vz/F) was greater than 1 L/kg, indicating that CTD from AEMC is rapidly and widely distributed in rat tissues after oral administration. Studies have also found that CTD from AEMC is mainly distributed in the liver and kidneys after oral administration. CTD is characterized in rat by its rapid distribution, and elimination in a short time for hepatic metabolism. This rapid hepatic uptake may be one of the reasons why the liver is a primary target organ for CTD toxicity (Duan et al., 2021).

Studies have shown that both oral and injectable preparations of CTD follow one-compartment model. The rapid t½_e_ of CTD is 0.63 h *in vivo*, which readily leads to peak and trough fluctuations in blood concentration when used clinically. This may be one of the reasons underlying the irritant nature of CTD. Additionally, the oral bioavailability of CTD in beagles has been reported to be low at 6.7%, likely due to its low solubility (Dang and Zhu, 2009).

Pharmacokinetic studies have provided basic data for the clinical safety of CTD, but there are still areas for improvement, such as insufficient assessment of species differences, dosage for special populations, and vigilance against data accuracy caused by errors in analytical methods.

## 7 Marketed drugs

Toxic mylabris has been combined with tonic drugs based on modern scientific techniques to develop, oral capsules (Kangsaidi capsules, China Food and Drug Administration approval number Z52020238) and injections (Aidi injection, China Food and Drug Administration approval number Z52020236). It represents a model for the application of contemporary preparations of mylabris and exemplifies the principle of “supporting the positive and dispelling the evil” in treating malignant tumors while using the TCM framework.

CTD is highly toxic and mostly used externally in the clinic settings. Based on the structure-activity relationship, a series of CTD derivatives, such as sodium cantharidinate and NCTD, have been developed to enhance the potency and attenuate the toxicity (Wang et al., 2019; Xiao et al., 2019; Zhu et al., 2020; Wu et al., 2021). These compounds have been approved by the China Food and Drug Administration for clinical use, and clinical studies have demonstrated their efficacy in patients. Table 4 summarizes the drug names, components/constituents, efficacy, and clinical uses of these marketed preparations. In addition, a summary of meta-analysis of Aidi injection and sodium cantharidinate Vitamin B6 Injection (DCVB6 injection) can lead to a more comprehensive and objective basis for the assessment of the therapeutic efficacy and safety of these drugs.

**TABLE 4:** Marketed products containing Mylabris or its related bioactive ingredients.

Drug	Components/constitutes	Efficacy	Clinical application
Kangsaidi capsules	1. Mylabris, 2. *Panax ginseng* C.A. Mey., 3. *Astragalus membranaceus* (Fisch.) Bunge., 4. *Acanthopanax senticosus* (Rupr. et Maxim.) Harms., 5. *Curcuma zedoaria*.*,* 6. *Scutellaria barbata* D. Don.*,* 7. *Curcuma aerugionosa*.*,* 8. *Cornus officinalis* Sieb. et Zucc., 9. Ursodeoxycholic acid., 10. *Glycyrrhiza uralensis* Fisch. ex DC.	Removing blood stasis, resolving static blood, attacking toxicity and corroding sores	Primary liver cancer, lung cancer, colorectal cancer, malignant lymphoma, gynecological malignancies
Aidi injection	1. Mylabris, 2. *Panax ginseng* C.A. Mey., 3. *Astragalus membranaceus*.*,* 4. *Eleutherococcus senticosus* (Rupr. and Maxim.) Maxim	Clearing heat and detoxifying, eliminating mass, and relieving swelling	Primary liver cancer, lung cancer, colorectal cancer, malignant lymphoma, gynecological malignancies
XuanShi YaoShui	1. Mylabris, 2. *Cortex cercidis*.*, 3. Zanthoxylum bungeanum* Maxim*.,*4. *Stemona tuberosa* Lour., *5. Saposhnikovia divaricata* (Turcz.) Schischk	Expelling wind and removing dampness, killing parasites, and relieving itching, antibacterial and anti-inflammatory effects	Dermatophytosis
Hupo Zhitong Gao	1. *Kaempferia galanga* L, 2. *Acorus calamus* var. angustatus Besser., 3. *Coptis chinensis* Franch., 4. Strychni Semen., 5. Mylabris, 6. *Clematis chinensis* Osbeck., 7. *Pinellia ternata* (Thunb.) Makino., 8. Venenum Bufonis., 9. Amber oil., 10. Clove basil oil., 11. Peppermint oil., 12. Star anise oil., 13. Cassia oil., 14. Borneolum syntheticum., 15. Camphorae	Activating blood circulation and resolving phlegm, reducing swelling, and dispersing nodules, promoting the flow of Qi and blood through the meridians, and relieving pain	Cancer pain, neuropathic pain, rheumatic pain, traumatic pain
Gan Ning tablets	1. Mylabris, 2. *Oryza sativa*.*,* 3. *Lithospermum erythrorhizon* Sieb. et Zucc	Clearing heat and detoxification, removing dampness, resolving blood stasis, and dispersing nodules	Acute and chronic hepatitis, prevention of hepatitis B related liver lesions, abnormal liver function
Cantharidine cream	Cantharidin	Antiviral	Verruca acuminata
Ycanth	Cantharidin	Antiviral	Molluscum contagiosum
Disodium cantharidinate and Vitamin B6 Injection	1. Disodium cantharidinate, 2. Vitamin B6	Antitumor	Advanced hepatocellular carcinoma, advanced lung cancer
Disodium cantharidinate injection	Disodium Cantharidinate	Antitumor	Primary liver cancer
Sodium demethylcantharidate injection	Sodium demethylcantharidate	Antitumor	Hepatocellular carcinoma, esophageal cancer, gastric and cardic cancer, lung cancer., as well as leukopenia, hepatitis, liver cirrhosis, and hepatitis B virus carriers
Demethylcantharidin Tablets	Norcantharidin	Antitumor	Hepatocellular carcinoma, esophageal cancer, gastric and cardiac cancer, leukopenia, hepatitis, liver cirrhosis and hepatitis B virus carriers

### 7.1 Aidi injection

Aidi injection is Chinese herbal injections belonging to the anti-cancer drugs categorys (CZ01) covered by the national basic medical insurance program for Chinese patent medicine in China (2004, 2009, 2017, 2019, 2023 version). It is the most competitive product in the field of cancer care in China in the *Report Science and Technology Competitiveness of Large Varieties of Chinese Medicine.* A meta-analysis of 3,300 patients revealed that Aidi injection is primarily used to treat lung, liver, and colon cancer. Its principal function is to enhance the survival of cancer patients, improve life quality and alleviate the adverse effects of chemotherapy and radiotherapy. This may be a contributing factor for the recommendation of Aidi injection as an adjuvant therapy to chemotherapy/radiotherapy in almost all of the included systematic reviews (Wang J. et al., 2018; Lan et al., 2021; Yang et al., 2022; Lu et al., 2024), in accordance with the national comprehensive cancer network guidelines.

### 7.2 DCVB6 injection

DCVB6 injection (China Food and Drug Administration approval number H20053,862) is a combination of sodium cantharidinate and vitamin B6 that is mainly used to treat HCC and non-small-cell lung cancer (NSCLC) (Zhu et al., 2020). A study evaluated the efficacy and safety of DCVB6 Injection in 104 patients with HCC and reported improvements in liver function, changes in tumor morphology and patient’s overall conditions (Shao et al., 2014). Furthermore, chemoradiotherapy regimens that are commonly used to treat HCC can often cause serious adverse effects. A meta-analysis evaluated its the feasibility as an alternative therapy to chemotherapy to evaluated survival, liver function, immune function, and quality of life (Zhu et al., 2020). A meta-analysis (19 trials, 1,428 patients with NSCLC) evaluated the combination of DCVB6 injection with chemotherapy versus chemotherapy alone and highlighted a reduction chemotherapy-induced side effects and improvement in clinical symptoms after using the combination (Wang et al., 2019). Another clinic trial (86 patients) has evaluated the improvement in the quality, and reduction of the side effects of chemotherapy in patients treated with DCVB6 compared with chemotherapy (Wang and Cui, 2014), providing strong clinical evidence for the use of DCVB6 in treating patients with neoplasms.

However, the clinical use of mylabris and its derivatives requires more high-quality clinical studies to be conducted and analyzed in line with the requirements for evidence-based medicine. Moreover, elucidation of its mechanism of action using modern molecular biology techniques is required, to better support its rational clinical application.

## 8 Conclusions and future prospects

Animal-derived medicines constitute a critical component of TCM, with documented therapeutic applications spanning millennia. The compendium of materia medica (*Ben Cao Gang Mu*), compiled by Li Shizhen during the Ming Dynasty, catalogs 1,892 medicinal substances, of which 444 agents are derived from animal sources. Similarly, the *Encyclopedia of Chinese Herbal Medicine* documents 5,767 TCM, including 740 animal-derived medicines, underscoring their enduring pharmacological and clinical relevance (Liu Y. et al., 2024). Contemporary studies have elucidated the mechanistic basis for their efficacy, particularly in antitumor, anti-inflammatory, and immunoregulation.

As an animal-derived TCM, mylabris holds significant medicinal value, yet its research and application remain inadequate. Regarding resources, the World Health Organization, in its Traditional Medicine Strategy (2014–2023), also emphasized that the development of TCM should be balanced with biodiversity conservation. The scarcity of mylabris and the overexploitation of wild populations pose a serious threat to its sustainable utilization. Regarding chemical composition, only a limited number of compounds have been isolated and characterized, and systematic investigations into the complex chemical basis of mylabris remain insufficient. Pharmacological studies have mainly focused on antitumor, and developed a series of clinical preparations, such as Aidi injection and DCVB6 Injection. However, the potential therapeutic effects recorded in ancient books on skin diseases, reproductive dysfunction, and digestive diseases have not yet been fully verified. Meanwhile, in agriculture, CTD exerts its broad spectrum insecticidal effects through gastric toxicity, contact effects, and anti-ingestion effects. It also has the potential to be a low residue, environmentally friendly insecticide, but is still in its early stages of development. In terms of toxicology, traditional records have suggested that it has contraindicated combination, such as its incompatibility with *Croton tiglium* L. and *Salvia miltiorrhiza* Bunge. Modern research has also confirmed that it can cause damage to multiple organs including the liver and kidneys, highlighting its safety risks. In terms of pharmacokinetics, preclinical studies have shown that CTD is primarily distributed in the liver and kidneys and exhibits low bioavailability. However, due to species differences and methodological limitations, there is a lack of clinical validation, which hinders the evaluation of safety and efficacy.

In the future, mylabris should be studied and developed from different levels. (1) artificial breeding and resource conservation strategies should be established to ensure the sustainable utilization of this medicinal material. (2) only a limited number of compounds have been identified, and a comprehensive analysis of the complex chemical composition, including small molecules, polysaccharides, lipids and other potential bioactive constituents, is still needed. Additionally, chemical fingerprint and quality control standards for mylabris should be established, along with quantitative methods for key active ingredients, to provide a foundation for clinical applications and international promotion. (3) by integrating traditional processing knowledge with modern pharmaceutical technologies, toxicity reduction and efficacy enhancement can be achieved through approaches such as structural modification, nanocarriers, liposomes, and other novel delivery systems (Xu et al., 2022). (4) Future pharmacological research on mylabris could extend beyond antitumor activity to explore other therapeutic areas, such as dermatological disorders or reproductive dysfunction, among others, guided by traditional records. In agriculture, mylabris also shows potential as an environmentally friendly biopesticide, suggesting promising directions for both medical and agricultural applications. (5) clinical pharmacokinetic studies and evidence based research should be strengthened to provide solid data support for rational drug use, safety testing, and international promotion. So, by integrating traditional wisdom with modern technology, mylabris is expected to realize safe and sustainable development.

## Glossary

AKT: Protein kinase B
ATG3: Autophagy-related 3
ATF4: Activating Transcription Factor 4
AUC: Area under the curve
BAG3: BCL2-associated athanogene-3
Bcl-2: B-cell lymphoma-2
Be: Beryllium
BRCA-1: Breast cancer 1
Ca: Calcium
CCAT1: Colon Cancer Associated Transcript 1
CDK1: Cyclin-dependent kinases 1
CDK2: Cyclin-dependent kinases 2
Cd: Cadmium
CHK1: Checkpoint Kinase 1
Cu: Copper
CTD: Cantharidin
COX: Cytochrome c oxidase
Cr: Chromium
Crb2: Growth factor receptor-bound protein 2
CSF2: Colony stimulating factor 2
CXCL8: C-X-C Motif Chemokine Ligand 8
DCVB6 injection: Sodium cantharidinate Vitamin B6 Injection
EGFR: Epidermal growth factor receptor
EphB4: PH Receptor B4
ER: Endoplasmic reticulum
ERK: Extracellular regulated protein kinases
FAK: Focal adhesion kinase
GM-CSF: Granulocyte-macrophage colony-stimulating factor
Glut1: Glucose transporter type 1
GRO: Growth-related oncogene
GRP78: Glucose regulatory protein 78
HCC: Hepatocellular carcinoma
Hg: Mercury
HSP70: Heat Shock Protein 70
IRE1α: Inositol-requiring enzyme 1α
JNK: C-Jun N-terminal kinase
KDM4A: Lysine Demethylase 4A
K_a_: Absorption rate constant
K_e_: Elimination rate constant
LC3: Microtubule-associated proteins light chain 3
LD50: Median lethal dose
LPS: Lipopolysaccharide
MACC1: Metastasis-associated in colon cancer 1
MAPK: Mitogen-activated protein kinase
MC: Molluscum contagiosum
MCT4: Monocarboxylate transporter 4
MDR: Multidrug resistance
MDM2: Mouse doubleminute 2 homolog
MEK: Mitogen-activated protein
Mg: Magnesium
MKK7: Mitogen-activated protein kinase 7
MMP: Matrix metalloproteinase
MMAC1: Mutated in multiple advanced cancer 1
Mn: Manganese
mTOR: Mammalian target of rapamycin
Na: Natrium
NCTD: Norcantharidin
Ni: Nickel
PARP: Poly ADP-ribose polymerase
Pb: Lead
PERK: Protein kinase R-like endoplasmic reticulum kinase
PI3K: Phosphatidylinositol 3-kinase
PKC: Protein kinase C
PTGS2: Prostaglandin-endoperoxide synthase 2
ROCK1: Rho associated coiled-coil containing protein kinase 1
Sn: Tin
TIMP2: Tissue inhibitor of metalloproteinase 2
t½_e_: Apparent elimination half-life
t½_a_: Half-life of apparent distribution
uPA: Urokinase-type plasminogen activator
VEGF: Vascular endothelial growth factor
Vz/F: Volume of distribution
WHO: World health organization
XBP-1: X-box binding protein 1
Zn: Zinc

## References

[B1] Braue A. Ross G. Varigos G. Kelly H. (2005). Epidemiology and impact of childhood molluscum contagiosum: a case series and critical review of the literature. Pediatr. Dermatol. 22 (4), 287–294. 10.1111/j.1525-1470.2005.22401.x 16060861

[B2] Cao W. D. Zhang Z. Y. Yang B. D. Zhang M. Z. Sun S. L. (2008). Inhibition of cantharidin and demethylcantharidin to seven phytopathogenic fungi. Acta. Phytophy Sin. (01), 63–68. 10.13802/j.cnki.zwbhxb.2008.01.008

[B3] Chai J. (2023). Study on the potential active components and mechanism of aidi injection in the treatment of primary hepatic carcinoma based on network pharmacology. Shandong: Shandong University of Traditional Chinese Medicine. master.

[B4] Chen Y. J. Tsai Y. M. Kuo C. D. Ku K. L. Shie H. S. Liao H. F. (2009). Norcantharidin is a small-molecule synthetic compound with anti-angiogenesis effect. Life. Sci. 85, 642–651. 10.1016/j.lfs.2009.09.003 19765597

[B5] Cheng W. Wang Y. Liu J. Li X. Yu M. Duan C. (2022). Hepatotoxicity of cantharidin is associated with the altered bile acid metabolism. J. Appl. Toxicol. 42, 970–980. 10.1002/jat.4267 34866203

[B6] Chun J. Park M. K. Ko H. Lee K. Kim Y. S. (2018). Bioassay-guided isolation of cantharidin from blister beetles and its anticancer activity through inhibition of epidermal growth factor receptor-mediated STAT3 and Akt pathways. J. Nat. Med. 72, 937–945. 10.1007/s11418-018-1226-6 30043217

[B7] Cui F. L. Li X. Ma Z. Q. Zhang Y. L. (2009). Safety evaluation of animal-origin pesticide cantharidin against some non-target organisms. J. Environ. Entomol. 31, 143–149. 10.3969/j.issn.1674-0858.2009.02.008

[B8] Dang Y. J. Zhu C. Y. (2009). Pharmacokinetics and bioavailability of cantharidin in beagle dogs. Zhongguo Zhong Yao Za Zhi 34, 2088–2091. 10.3321/j.issn:1001-5302.2009.16.021 19938553

[B9] Deng L. P. Dong J. Cai H. Wang W. (2013). Cantharidin as an Antitumor agent: a retrospective review. Curr. Med. Chem. 20 (2), 159–166. 10.2174/092986713804806711 23210849

[B10] Deng Y. Y. Zhang W. Li N. P. Lei X. P. Gong X. Y. Zhang D. M. (2017). Cantharidin derivatives from the medicinal insect Mylabris phalerata. Tetrahedron 73, 5932–5939. 10.1016/j.tet.2017.08.034

[B11] Díaz-Navarro M. Bolívar P. Andrés M. F. Gómez-Muñoz M. T. Martínez-Díaz R. A. Valcárcel F. (2021). Antiparasitic effects of potentially toxic beetles (Tenebrionidae and Meloidae) from steppe zones. Toxins 13, 489. 10.3390/toxins13070489 34357960 PMC8310226

[B12] Du Y. F. (2019). The mechanism of cantharidin induced apoptosis in breast cancer cells. Nanjing: China Medical University. master.

[B13] Duan C. Cheng W. Chen Q. Li X. Zhang J. (2021). Pharmacokinetics and tissue distribution of cantharidin after oral administration of aqueous extracts from mylabris in rats. Biomed. Chromatogr. 35, e5172. 10.1002/bmc.5172 33982312

[B14] Efferth T. Li P. C. H. Konkimalla V. S. B. Kaina B. (2007). From traditional Chinese medicine to rational cancer therapy. Trends. Mol. Med. 13, 353–361. 10.1016/j.molmed.2007.07.001 17644431

[B15] Eisner T. Johnessee J. S. Carrel J. Hendry L. B. Meinwald J. (1974). Defensive use by an insect of a plant resin. Science 184, 996–999. 10.1126/science.184.4140.996 4207808

[B16] Fang L. Du G. (2018). The historical cognition and evaluation of mylabris toxicity. Pharmacol. Clin. Chin. Mater. Medica. 34, 150–152. 10.13412/j.cnki.zyyl.2018.05.037

[B17] Gu X. D. Xu L. L. Zhao H. Gu J. Z. Xie X. H. (2017). Cantharidin suppressed breast cancer MDA-MB-231 cell growth and migration by inhibiting MAPK signaling pathway. Braz. J. Med. Biol. Res. 50, e5920. 10.1590/1414-431x20175920 28678918 PMC5496155

[B18] Guo F. Tian X. Y. Xiong X. Q. Yuan Z. W. Zhang L. Yuan Y. J. (2023). Regulation of platelet function by cantharidin via PI3K/Akt/PKC pathway. Chin. Pharmacol. Bull. 39, 1248–1255. 10.12360/CPB202209015

[B19] He T. M. Zhang J. Y. Liu L. Li X. F. (2022). Research advances of mylabris-induced hepatorenal toxicity in recent years. Chin. J. Mod. Appl. Pharm. 39, 3310–3315. 10.13748/j.cnki.issn1007-7693.2022.24.020

[B20] He T. M. Chen K. Xiong L. J. Lin K. X. Lu D. Y. Li X. F. (2024). Liver injury induced by cantharidin through endoplasmic reticulum stress, autophagy, and apoptosis in rat. Chin. J. Mod. Appl. Pharm. 41, 156–165. 10.13748/j.cnki.issn1007-7693.20232191

[B21] Hou B. C. Wang X. W. He Z. J. Liu H. Y. (2024). Integrative approach using network pharmacology, bioinformatics, and experimental methods to explore the mechanism of cantharidin in treating colorectal cancer. Naunyn Schmiedeb. Arch. Pharmacol. 397, 6745–6761. 10.1007/s00210-024-03041-7 38507104

[B22] Hsia T. C. Yu C. C. Hsu S. C. Tang N. Y. Lu H. F. Huang Y. P. (2014). Cantharidin induces apoptosis of H460 human lung cancer cells through mitochondria-dependent pathways. Int. J. Oncol. 45, 245–254. 10.3892/ijo.2014.2428 24818581

[B23] Hsia T. C. Lin J. H. Hsu S. C. Tang N. Y. Lu H. F. Wu S. H. (2015). Cantharidin induces DNA damage and inhibits DNA repair-associated protein levels in NCI-H460 human lung cancer cells. Environ. Toxicol. 30, 1135–1143. 10.1002/tox.21986 24639390

[B24] Hsia T. C. Yu C. C. Hsiao Y. T. Wu S. H. Bau D. T. Lu H. F. (2016). Cantharidin impairs cell migration and invasion of human lung cancer NCI-H460 cells *via* UPA and MAPK signaling pathways. Anticancer Res. 36, 5989–5997. 10.21873/anticanres.11187 27793925

[B25] Hsieh C. H. Chao K. S. C. Liao H. F. Chen Y. J. (2013). Norcantharidin, derivative of cantharidin, for cancer stem cells. *Evid. Based* . Complement. Altern. Med. 2013, 1–11. 10.1155/2013/838651 PMC3773992 24073010

[B26] Hsieh F. S. Hung M. H. Wang C. Y. Chen Y. L. Hsiao Y. J. Tsai M. H. (2017). Inhibition of protein phosphatase 5 suppresses non-small cell lung cancer through AMP-Activated kinase activation. Lung Cancer 112, 81–89. 10.1016/j.lungcan.2017.07.040 29191605

[B27] Huang W. W. Ko S. W. Tsai H. Y. Chung J. G. Chiang J. H. Chen K. T. (2011). Cantharidin induces G2/M phase arrest and apoptosis in human colorectal cancer colo 205 cells through inhibition of CDK1 activity and caspase-dependent signaling pathways. Int. J. Oncol. 38, 1067–1073. 10.3892/ijo.2011.922 21271215

[B28] Huang Z. Wang Y. Zhang Y. (2015). Lethal and sublethal effects of cantharidin on development and reproduction of Plutella xylostella (lepidoptera: plutellidae). J. Econ. Entomol. 108 (3), 1054–1064. 10.1093/jee/tov057 26470229

[B29] Karras D. J. Farrell S. E. Harrigan R. A. Henretig F. M. Gealt L. (1996). Poisoning from “Spanish fly” (Cantharidin). Am. J. Emerg. Med. 14, 478–483. 10.1016/S0735-6757(96)90158-8 8765116

[B30] Kim J. A. Kim Y. Kwon B. M. Han D. C. (2013). The natural compound cantharidin induces cancer cell death through inhibition of heat shock protein 70 (HSP70) and Bcl-2-associated athanogene domain 3 (BAG3) expression by blocking heat shock factor 1 (HSF1) binding to promoters. J. Biol. Chem. 288, 28713–28726. 10.1074/jbc.M113.488346 23983126 PMC3789968

[B31] Lan H. Y. An P. Liu Q. P. Chen Y. Y. Yu Y. Y. Luan X. (2021). Aidi injection induces apoptosis of hepatocellular carcinoma cells through the mitochondrial pathway. J. Ethnopharmacol. 274, 114073. 10.1016/j.jep.2021.114073 33794335

[B32] Le A. P. Zhang L. L. Liu W. Shi Y. F. (2016). Cantharidin inhibits cell proliferation and induces apoptosis through G2/M phase cell cycle arrest in hepatocellular carcinoma stem cells. Oncol. Rep. 35, 2970–2976. 10.3892/or.2016.4684 26986084

[B33] Li J. (2023). The effect of cantharides on bone marrow cells in mouse and its regulatory mechanism. Henan: Henan University. master. 10.27114/d.cnki.ghnau.2021.000858

[B34] Li Y. K. Chen Z. (2025). Research progress on processing history evolution, chemical constituents and pharmacological action of Chinese blister beetle. Chin. Arch. Tradit. Chin. Med. 43 (05), 202–209. 10.13193/j.issn.1673-7717.2025.05.037 38621894

[B35] Li X. F. Chen X. S. Wang X. M. Hou X. H. (2007). Contents and existing forms of cantharidin in Meloidae(Coleoptera). Acta Entomol. Sin., 750–754. 10.16380/j.kcxb.2007.07.001

[B36] Li T. Kang G. Wang T. Huang H. (2018). Tumor angiogenesis and anti-angiogenic gene therapy for cancer. Oncol. Lett. 16, 687–702. 10.3892/ol.2018.8733 29963134 PMC6019900

[B37] Li W. J. Xia H. Jiang H. Xu C. W. Qiu L. (2020). Effect of cantharidin on neointimal hyperplasia after carotid balloon injury in rats. Chin. Circ. J. 35, 293–298. 10.3969/j.issn.1000-3614.2020.03.012

[B38] Li N. Miao M. S. Bai L. (2022). Clinical toxicity mechanism and rescue measures of highly toxic traditional Chinese medicine. Chin. Arch. Tradit. Chin. Med. 37, 659–664.

[B39] Li S. Duan X. Zhang Y. Zhao C. Yu M. Li X. (2024). Lipidomics reveals serum lipid metabolism disorders in CTD-induced liver injury. Bmc. Pharmacol. Toxicol. 25 (1), 10. 10.1186/s40360-024-00732-y 38225635 PMC10790540

[B40] Li. K. M. Li J. J. Wan L. Cheng Y. X. (2023). Five new cantharidin derivatives from the insect *Mylabris cichorii* L. and their potential against kidney fibrosis *in vitro* . Basel Switz. 28 (6), 2822. 10.3390/molecules28062822 36985794 PMC10056085

[B41] Li S. Wu X. Fan G. Du K. Deng L. (2023). Exploring cantharidin and its analogues as anticancer agents: a review. Curr. Med. Chem. 30, 2006–2019. 10.2174/0929867330666221103151537 36330637

[B42] Liu X. S. Zhang Y. F. Guo Z. M. Wang Y. (2013). Chemical constituents of Ethyl acetate fraction of Mylabris phalerata. J. Mt. Agric. Biol. 32, 187–188. 10.15958/j.cnki.sdnyswxb.2013.02.001

[B43] Liu T. T. Wang J. Q. Zhao L. J. (2018). Influence of cantharidin on proliferation and apoptosis of malignant melanoma A375 cells and its mechanism. J. QIqihar. Med. Univ. 39, 874–878. 10.3969/j.issn.1002-1256.2018.08.002

[B44] Liu H. B. Shi Y. C. Ren Y. Wan D. G. (2019). Overview of the research on the national medicinal research of cantharides and its related species. Chin. Tradit. Pat. Med. 41, 691–694. 10.3969/j.issn.1001-1528.2019.03.046

[B45] Liu F. Duan C. Zhang J. Li X. (2020). Cantharidin-induced LO2 cell autophagy and apoptosis *via* endoplasmic reticulum stress pathway *in vitro* . J. Appl. Toxicol. 40, 1622–1635. 10.1002/jat.4022 32638414

[B46] Liu R. Yang C. Yang X. Yu J. Tang W. (2024). Network toxicology, molecular docking technology, and experimental verification revealed the mechanism of cantharidin-induced testicular injury in mice. Toxicol. Appl. Pharmacol. 486, 116921. 10.1016/j.taap.2024.116921 38582374

[B47] Liu Y. Song Y. G. Zhao R. S. Miao M. S. (2024). Characteristics and analysis of animal-derived drugs in 2020 edition of Chinese pharmacopoeia. Chin. J. Exp. Tradit. Med. From. 30, 218–224. 10.13422/j.cnki.syfjx.20231712

[B48] Lou T. T. Du J. Chen X. S. Li S. W. (2014). Inhibitory effect of cantharidin on proliferation of prostate cancer PC- 3 cells. J. Mt. Agric. Biol. 33, 61–63+68. 10.15958/j.cnki.sdnyswxb.2014.02.018

[B49] Lou F. M. Li X. F. Liu Y. (2018). Study on the differences of metal elements in cantharides from different regions and varieties. J. South China Normal Univ. Sci. Ed. 50 (04), 33–36. 10.6054/j.jscnun.2018061

[B50] Lu S. Huang J. Zhang J. Wu C. Huang Z. Tao X. (2024). The anti-hepatocellular carcinoma effect of aidi injection was related to the synergistic action of cantharidin, formononetin, and isofraxidin through BIRC5, FEN1, and EGFR. J. Ethnopharmacol. 319, 117209. 10.1016/j.jep.2023.117209 37757991

[B51] Mo R. Y. Sun N. N. Peng R. (2014). Study on preferred food of adult Mylabris phalerata in different geographical populations. China J. Chin. Mater. Medica. 39, 4293–4296. 25850255

[B52] Mo L. Zhang X. Shi X. Wei L. Zheng D. Li H. (2018). Norcantharidin enhances antitumor immunity of GM ‐ CSF prostate cancer cells vaccine by inducing apoptosis of regulatory T cells. Cancer. Sci. 109, 2109–2118. 10.1111/cas.13639 29770533 PMC6029826

[B53] Moed L. Shwayder T. A. Chang M. W. (2001). Cantharidin revisited: a blistering defense of an ancient medicine. Arch. Dermatol. 137, 1357–1360. 10.1001/archderm.137.10.1357 11594862

[B54] Moye V. Cathcart S. Burkhart C. N. Morrell D. S. (2013). Beetle juice: a guide for the use of cantharidin in the treatment of molluscum contagiosum. Dermatol. Ther. 26, 445–451. 10.1111/dth.12105 24552407

[B55] Nazim U. M. Yin H. Park S. Y. (2020). Downregulation of c FLIP and upregulation of DR 5 by cantharidin sensitizes TRAIL mediated apoptosis in prostate cancer cells *via* autophagy flux. Int. J. Mol. Med. 46, 280–288. 10.3892/ijmm.2020.4566 32319535 PMC7255450

[B56] Pan Y. Zheng Q. Ni W. Wei Z. Yu S. Jia Q. (2019). Breaking glucose transporter 1/Pyruvate kinase M2 glycolytic loop is required for cantharidin inhibition of metastasis in highly metastatic breast cancer. Front. Pharmacol. 10, 590. 10.3389/fphar.2019.00590 31178738 PMC6544055

[B57] Pan M. S. Cao J. Fan Y. Z. (2020). Insight into norcantharidin, a small-molecule synthetic compound with potential multi-target anticancer activities. Chin. Med. 15, 55. 10.1186/s13020-020-00338-6 32514288 PMC7260769

[B58] Pradhan R. N. Shrestha B. Lee Y. (2024). Avoiding cantharidin through ionotropic receptors. J. Hazard. Mater. 466, 133497. 10.1016/j.jhazmat.2024.133497 38278077

[B59] Qi L. W. Zhou J. L. Hao H. P. Li H. J. Wen X. D. Chen J. (2010). Biological-chemical profiling of *in vitro* and *in vivo* bioactive compounds from traditional Chinese medicines with holistic views. J. China. Pharm. Univ. 41, 195–202.

[B60] Qiu L. Q. Xia H. Jiang H. Li W. J. Wu G. Luo Q. (2019a). Inhibitory effect of cantharidin on rat vascular smooth muscle cells proliferation and migration by blocking NF-κB signaling pathway. Chin. Circ. J. 34, 503–510. 10.3969/j.issn.1000-3614.2019.05.015

[B61] Qiu L. Q. Xu C. W. Li W. J. Jiang H. Wu G. Luo Q. (2019b). Mechanism of cantharidin underlying proliferation and migration of vascular smooth muscle cells induced by PDGF-BB. Chin. J. Geriatr. Heart. Brain. Vessel. Dis. 21 (01), 58–62. 10.3969/j.issn.1009-0126.2019.01.015

[B62] Ratcliffe N. A. Mello C. B. Garcia E. S. Butt T. M. Azambuja P. (2011). Insect natural products and processes: new treatments for human disease. Insect biochem. Mol. Biol. 41, 747–769. 10.1016/j.ibmb.2011.05.007 21658450

[B63] Ren Y. Liu H. B. Deng D. Mo R. Y. Wu F. M. (2020). Historical evolution in processing and some problems in modern research of mylabris. Chin. Tradit. Herb. Drugs. 51, 4082–4091. 10.7501/j.issn.0253-2670.2020.15.029

[B64] Safenraiter M. E. Soldini M. P. C. Río M. G. (2024). Cantharidin: a multiporpuse beetlejuice. Neotrop. Entomol. 53, 964–971. 10.1007/s13744-024-01164-3 38750300

[B65] Schöpe P. C. Zinnow V. Ishfaq M. A. Smith J. Herrmann P. Shoemaker R. H. (2023). Cantharidin and its analogue norcantharidin inhibit metastasis-inducing genes S100A4 and MACC1. Int. J. Mol. Sci. 24, 1179. 10.3390/ijms24021179 36674695 PMC9866560

[B66] Shao H. Hong G. Luo X. (2014). Evaluation of sodium cantharidinate/vitamin B6 in the treatment of primary liver cancer. J. Cancer Res. Ther. 10, 75–78. 10.4103/0973-1482.139770 25207897

[B67] Shen B. He P. J. Shao C. L. (2013). Norcantharidin induced DU145 cell apoptosis through ROS-mediated mitochondrial dysfunction and energy depletion. PLOS. ONE. 8, e84610. 10.1371/journal.pone.0084610 24367681 PMC3868658

[B68] Song X. X. (2014). Study on quality control methods and toxicokinetics of mylabris and its preparations. Shenyang: Shenyang Pharmaceutical University. master.

[B69] Song M. Wang X. Luo Y. Liu Z. Tan W. Ye P. (2020). Cantharidin suppresses gastric cancer cell migration/invasion by inhibiting the PI3K/Akt signaling pathway *via* CCAT1. Chem. Biol. Interact. 1 (317), 108939. 10.1016/j.cbi.2020.108939 31945315

[B70] Su C. C. Liu S. H. Lee K. I. Huang K. T. Lu T. H. Fang K. M. (2015). Cantharidin induces apoptosis through the Calcium/PKC-Regulated endoplasmic reticulum stress pathway in human bladder cancer cells. Am. J. Chin. Med. 43, 581–600. 10.1142/S0192415X15500366 25967669

[B71] Su C. C. Lee K. I. Chen M. K. Kuo C. Y. Tang C. H. Liu S. H. (2016). Cantharidin induced oral squamous cell carcinoma cell apoptosis *via* the JNK-regulated mitochondria and endoplasmic reticulum stress-related signaling pathways. PLOS. ONE. 11, e0168095. 10.1371/journal.pone.0168095 27930712 PMC5145211

[B72] Sui T. T. Tian L. C. Zhang X. Ma Q. X. Feng Y. Y. Hu X. W. (2018). Synergistic effect of cantharidin and pemetrexed on HCT116 colorectal cancer cells. Chin. J. Exp. Tradit. Med. Formulae 24, 43–48. 10.13422/j.cnki.syfjx.20181618

[B73] Sun X. Cai X. Yang J. Chen J. Guo C. Cao P. (2016). Cantharidin overcomes imatinib resistance by depleting BCR-ABL in chronic myeloid leukemia. Mol. Cells. 39, 869–876. 10.14348/molcells.2016.0023 27989101 PMC5223104

[B74] Vakharia P. P. Chopra R. Silverberg N. B. Silverberg J. I. (2018). Efficacy and safety of topical cantharidin treatment for molluscum contagiosum and warts: a systematic review. Am. J. Clin. Dermatol. 19, 791–803. 10.1007/s40257-018-0375-4 30097988

[B75] Viallard C. Larrivée B. (2017). Tumor angiogenesis and vascular normalization: alternative therapeutic targets. Angiogecnesis 20, 409–426. 10.1007/s10456-017-9562-9 28660302

[B76] Wang Z. J. (2014). Research on insecticidal technology of the cantharidin and its derivatives. Shanxi: Northwest A&F University. master.

[B77] Wang B. Cui J. (2014). Treatment of mid-late stage NSCLC using sodium cantharidinate/vitamin B6/GP regimen in clinic. J. Cancer. Res. Ther. 10, 79–C81. 10.4103/0973-1482.139771 25207898

[B78] Wang T. Liu J. Xiao X. Q. (2015). Cantharidin inhibits angiogenesis by suppressing VEGF-Induced JAK1/STAT3, ERK and AKT signaling pathways. Arch. Pharm. Res. 38, 282–289. 10.1007/s12272-014-0383-8 24733674

[B79] Wang G. Dong J. Deng L. (2018a). Overview of cantharidin and its analogues. Curr. Med. Chem. 25, 2034–2044. 10.2174/0929867324666170414165253 28413963

[B80] Wang J. Li G. Yu L. Mo T. Wu Q. Zhou Z. (2018b). Aidi injection plus platinum-based chemotherapy for stage IIIB/IV non-small cell lung cancer: a meta-analysis of 42 RCTs following the PRISMA guidelines. J. Ethnopharmacol. 221, 137–150. 10.1016/j.jep.2018.04.013 29655852

[B81] Wang Z. Feng F. Wu Q. Jin Y. Gu C. Xu Y. (2019). Disodium cantharidinate and vitamin B6 injection adjunct with platinum-based chemotherapy for the treatment of advanced non-small-cell lung cancer: a meta-analysis. Evid. Based Complement. Altern. Med. 2019, 9386273–14. 10.1155/2019/9386273 30992710 PMC6434307

[B82] Wang J. Bao L. Du J. Y. Zhang L. F. Wang H. S. (2022). Action mechanism and experimental verification of WBGSF in treating DN based on network pharmacology. Inf. Tradit. Chin. Med. 39, 21–26. 10.19656/j.cnki.1002-2406.20221205

[B83] Wei C. Deng X. Gao S. Wan X. Chen J. (2022). Cantharidin inhibits proliferation of liver cancer by inducing DNA damage *via* KDM4A-Dependent histone H3K36 demethylation. Evid. Based Complement. Altern. Med. 2022, 2197071–2197079. 10.1155/2022/2197071 35860003 PMC9293552

[B84] West E. Krychman M. (2015). Natural aphrodisiacs—A review of selected sexual enhancers. Sex. Med. Rev. 3, 279–288. 10.1002/smrj.62 27784600

[B85] Wu W. Su M. Li T. Wu K. Wu X. Tang Z. (2015). Cantharidin-induced liver injuries in mice and the protective effect of vitamin C supplementation. Int. Immunopharmacol. 28, 182–187. 10.1016/j.intimp.2015.06.003 26071218

[B86] Wu L. Deng C. Zhang H. Weng J. Wu Y. Zeng S. (2021). Efficacy and safety of docetaxel and sodium cantharidinate combination vs. either agent alone as second-line treatment for advanced/metastatic NSCLC with wild-type or unknown EGFR status: an open-Label, randomized controlled, prospective, multi-center phase III trial (Cando-L1). Front. Oncol. 11, 769037. 10.3389/fonc.2021.769037 34976813 PMC8715707

[B87] Xiao W. J. Dai B. Zhu Y. Ye D. W. (2016). Norcantharidin induces autophagy-related prostate cancer cell death through Beclin-1 upregulation by miR-129-5p suppression. Tumor Biol. 37, 15643–15648. 10.1007/s13277-015-4488-6 26638170

[B88] Xiao Z. Wang C. Tan Z. Hu S. Chen Y. Zhou M. (2019). Clinical efficacy and safety of sodium cantharidinate plus chemotherapy in non-small-cell lung cancer: a systematic review and meta-analysis of 38 randomized controlled trials. J. Clin. Pharm. Ther. 44, 23–38. 10.1111/jcpt.12761 30229971

[B89] Xiao Y. Liu R. Tang W. Yang C. (2024). Cantharidin-induced toxic injury, oxidative stress, and autophagy attenuated by Astragalus polysaccharides in mouse testis. Reprod. Toxicol. 123, 108520. 10.1016/j.reprotox.2023.108520 38056682

[B90] Xu M. D. (2019). The combination of cantharidin and anti-angiogenic therapeutics presents synergistic antitumor effects against pancreatic cancer. Jiangsu: Soochow University. master.

[B91] Xu M. D. Liu S. L. Zheng B. B. Wu J. Wu M. Y. Zhang Y. (2018). The radiotherapy-sensitization effect of cantharidin: mechanisms involving cell cycle regulation, enhanced DNA damage, and inhibited DNA damage repair. Pancreatology 18, 822–832. 10.1016/j.pan.2018.08.007 30201439

[B92] Xu Y. Wang M. Ning S. Yang Z. Zhou L. Xia X. (2022). Development of glycyrrhetinic acid and folate modified cantharidin loaded solid lipid nanoparticles for targeting hepatocellular carcinoma. Mol. Basel. Switz. 27, 6786. 10.3390/molecules27206786 36296377 PMC9610810

[B93] Yan J. Deng X. L. Ma S. Q. Li Y. H. Gao Y. M. Shi G. T. (2023). Cantharidin suppresses hepatocellular carcinoma development by regulating EZH2/H3K27me3-dependent cell cycle progression and antitumour immune response. BMC Complement. Med. Ther. 18, 160. 10.1186/s12906-023-03975-0 37202806 PMC10193799

[B94] Yang M. Shen C. Zhu S. Zhang Y. Jiang H. Bao Y. (2022). Chinese patent medicine aidi injection for cancer care: an overview of systematic reviews and meta-analyses. J. Ethnopharmacol. 282, 114656. 10.1016/j.jep.2021.114656 34551361

[B95] Yang T. Yu R. Cheng C. Huo J. Gong Z. Cao H. (2023). Cantharidin induces apoptosis of human triple negative breast cancer cells through mir-607-mediated downregulation of EGFR. J. Transl. Med. 21, 597. 10.1186/s12967-023-04483-y 37670360 PMC10481602

[B96] Yin Y. Jin G. (2010). Biosynthesis transfer and biological function of cantharidin in blister beetles. Acta. entamologica. Sin. 53, 1305–1313. 10.16380/j.kcxb.2010.11.010

[B97] Yu Y. L. Zhang Y. Y. Zhang J. Guan C. Liu L. Ren L. (2020a). Cantharidin‐induced acute hepatotoxicity: the role of TNF‐α, IKK‐α, Bcl‐2, bax and caspase3. J. Appl. Toxicol. 40, 1526–1533. 10.1002/jat.4003 32627230

[B98] Yu Z. Li L. Wang C. He H. Liu G. Ma H. (2020b). Cantharidin induces apoptosis and promotes differentiation of AML cells through nuclear receptor Nur77-Mediated signaling pathway. Front. Pharmacol. 11, 1321. 10.3389/fphar.2020.01321 32982739 PMC7485522

[B99] Zeng Y. B. Liu X. L. Li C. J. Zhou X. Zhang X. M. Yang Y. (2016). Chemical constituents from Mylabris phalerata and their cytotoxic activity *in vitro* . China J. Chin. Mater. Medica. 41, 859–863. 10.4268/cjcmm20160516 28875639

[B100] Zeng B. Chen X. Zhang L. Gao X. Gui Y. (2024). Norcantharidin in cancer therapy – a new approach to overcoming therapeutic resistance: a review. Med. Baltim. 103, e37394. 10.1097/MD.0000000000037394 38428865 PMC10906652

[B101] Zhang H. (2021). Effect of cantharidin on radiosensitivity of lung adenocarcinoma A-549 cells. Inner Mongolia: Inner Mongolia Medical University. master.

[B102] Zhang Y. Y. (2022). Experimental research on toxicological pathology and molecular biology in cardiotoxicity of acute cantharidin poisoning in sprague dawley rats. Zhejiang: Huazhong University of Science and Technology. master.

[B103] Zhang H. Yan X. (2015). Cantharidin modulates the E2F1/MCM7-miR-106b-93/p21-PTEN signaling axis in MCF-7 breast cancer cells. Oncol. Lett. 10, 2849–2855. 10.3892/ol.2015.3681 26722252 PMC4665410

[B104] Zhang Z. Y. Yuan F. Zhang X. (2000). Effect of catharidin on the digestive enymes and esterases of diamondback moth larva. Acta. phytophylacica. Sin., 355–358. 10.13802/j.cnki.zwbhxb.2000.04.013

[B105] Zhang C. Chen Z. Zhou X. Xu W. Wang G. Tang X. (2014). Cantharidin induces G2/M phase arrest and apoptosis in human gastric cancer SGC-7901 and BGC-823 cells. Oncol. Lett. 8, 2721–2726. 10.3892/ol.2014.2611 25364455 PMC4214476

[B106] Zhang Y. Zhou X. Zhang J. Guan C. Liu L. (2018). Cantharides poisoning: a retrospective analysis from 1996 to 2016 in China. Regul. Toxicol. Pharmacol. 96, 142–145. 10.1016/j.yrtph.2018.05.007 29753762

[B107] Zhang Y. Yu Y. Zhang J. Guan C. Liu L. Ren L. (2020). Biomarkers of myocardial injury in rats after cantharidin poisoning: application for postmortem diagnosis and estimation of postmortem interval. Sci. Rep. 10, 12069. 10.1038/s41598-020-69118-4 32694590 PMC7374104

[B108] Zhang Y. Y. Yu Y. L. Zhang J. Guan C. H. Liang L. Liang R. (2020). Molecular biomarkers of cantharidin‐induced cardiotoxicity in sprague‐dawley rats: troponin T, vascular endothelial growth factor and hypoxia inducible factor‐1α. J. Appl. Toxicol. 40, 1153–1161. 10.1002/jat.3974 32162354

[B109] Zhang Y. Y. Yu Y. L. Zhang J. Guan C. H. Ren L. Liu L. (2020). Research progress on multiple organ damage and mechanism of cantharidin poisoning. J. Forensic. Med. 36, 545–548. 10.12116/j.issn.1004-5619.2020.04.021 33047541

[B110] Zheng L. H. Bao Y. L. Wu Y. Yu C. L. Meng X. Li Y. X. (2008). Cantharidin reverses multidrug resistance of human hepatoma HepG2/ADM cells *via* down-regulation of P-glycoprotein expression. Cancer. Lett. 272, 102–109. 10.1016/j.canlet.2008.06.029 18703276

[B111] Zheng D. Sha Q. M. Wang J. Q. Liu Z. M. Wan X. F. Wang G. C. (2015). Effect of Norcantharidin on Hematopoietic Function in Leucopenia Model Rat Induced by Cyclophosphamide. J. Exp. Hematol. 23, 826–831. 10.7534/j.issn.1009-2137.2015.03.043 26117044

[B112] Zhongyoo (2012). Jianding. Available online at: http://www.zhongyoo.com/jianding/3004.html (Accessed March 16, 2025).

[B113] Zhu M. Liu X. Zhou C. Li J. (2020). Effect of sodium cantharidinate/vitamin B6 injection on survival, liver function, immune function, and quality of life in patients with hepatocellular carcinoma: protocol for a meta-analysis. Med. Baltim. 99, e21952. 10.1097/MD.0000000000021952 32846865 PMC7447480

